# Ultrasound-Assisted Recovery and Biological Evaluation of an Astaxanthin-Containing Extract from *Chara corallina*

**DOI:** 10.3390/antiox15091159

**Published:** 2026-09-11

**Authors:** Natthrit Roekngam, Wanninee Chankaew, Suwichak Chaisit, Sonsawan Kongpuckdee, Sunisa Khongthong

**Affiliations:** 1 Faculty of Veterinary Science, Rajamangala University of Technology Srivijaya, Nakhon Si Thammarat 80240, Thailand; natthrit.r@rmutsv.ac.th; 2 Faculty of Agriculture, Rajamangala University of Technology Srivijaya, Nakhon Si Thammarat 80110, Thailand; wanninee.c@rmutsv.ac.th; 3 Department of Chemical Engineering and Pharmaceutical Chemistry, School of Engineering and Technology, Walailak University, Nakhon Si Thammarat 80160, Thailand; suwichak.ch@mail.wu.ac.th; 4 Faculty of Health and Sports Science, Thaksin University, Pa Phayom, Phatthalung 93210, Thailand; sonsawan.k@tsu.ac.th

**Keywords:** astaxanthin, *Chara corallina*, ultrasound-assisted extraction, oxidative stress, photoaging, antioxidant, cosmeceutical, carotenoid

## Abstract

Oxidative stress and chronic inflammation are major contributors to skin photoaging, highlighting the need for natural antioxidants with multifunctional bioactivities. This study developed an ultrasound-assisted extraction (UAE) strategy to recover an astaxanthin-rich extract from the underutilized freshwater macroalga *Chara corallina* and evaluated its antioxidant, anti-inflammatory, and anti-photoaging properties. Different solvent systems were evaluated for extraction efficiency, and astaxanthin-equivalent recovery was quantified by high-performance liquid chromatography with photodiode array detection (HPLC–PDA). Antioxidant activity was evaluated using the DPPH radical scavenging assay, whereas biological activities were assessed in human dermal fibroblasts and RAW264.7 macrophages using cell viability assays, quantitative real-time PCR, and extracellular matrix-related enzyme inhibition assays. The selected solvent system (48% ethanol in ethyl acetate) provided the highest astaxanthin-equivalent recovery (0.2598 ± 0.0086% *w*/*w*). The selected CCE exhibited DPPH radical-scavenging activity, modulated antioxidant- and inflammation-associated gene expression, increased *COL1A2* mRNA expression, and inhibited collagenase, elastase, and hyaluronidase within the evaluated concentration range. HPLC–PDA analysis revealed a chromatographic component with retention-time and UV–visible spectral characteristics corresponding to those of an authentic astaxanthin reference standard; however, comprehensive structural and phytochemical characterization was not performed. Because CCE is a chemically complex extract, the observed biological responses cannot be attributed exclusively to astaxanthin. Overall, these findings provide an initial basis for further investigation of *C. corallina* as an underexplored freshwater source of carotenoid-containing bioactive extracts rather than establishing it as a commercially competitive source of natural astaxanthin.

## 1. Introduction

Skin aging is a multifactorial biological process driven by intrinsic aging and extrinsic environmental factors, particularly chronic exposure to ultraviolet (UV) radiation. Among these, photoaging is the predominant cause of premature skin deterioration and is characterized by wrinkle formation, loss of skin elasticity, collagen degradation, and impaired skin barrier function. These pathological changes are closely associated with persistent oxidative imbalance, chronic low-grade inflammation, and progressive disruption of extracellular matrix (ECM) homeostasis, ultimately leading to structural and functional deterioration of the dermis [1,2]. Consequently, increasing attention has been directed toward identifying natural bioactive compounds capable of simultaneously enhancing antioxidant defenses, modulating inflammatory responses, and preserving extracellular matrix integrity. Such multifunctional activities are considered highly desirable for the development of next-generation cosmeceutical ingredients and skin-protective formulations [1,3].

Among naturally occurring carotenoids, astaxanthin has attracted considerable interest because of its potent antioxidant, anti-inflammatory, and skin-protective properties. In addition to its direct radical-scavenging activity, astaxanthin has been reported to enhance endogenous antioxidant defense systems by regulating antioxidant genes and enzymes, including superoxide dismutase (SOD), glutathione peroxidase (GPx), and catalase (CAT), while modulating inflammation-associated signaling pathways [1,4]. Furthermore, astaxanthin has been shown to support collagen homeostasis, inhibit extracellular matrix-degrading enzymes, and maintain dermal fibroblast function, thereby contributing to the preservation of skin structure and elasticity during the aging process [2,5]. Owing to these multifunctional biological activities, astaxanthin has emerged as one of the most promising natural ingredients for functional foods, nutraceuticals, pharmaceuticals, and cosmeceutical products [3,6].

Commercial astaxanthin is predominantly produced from the microalga *Haematococcus pluvialis*, which is widely recognized as one of the richest natural sources of this high-value carotenoid [4,7]. Nevertheless, large-scale production of natural astaxanthin from *H. pluvialis* remains associated with substantial cultivation and downstream-processing requirements, including controlled biomass production, induction of astaxanthin accumulation, disruption of the resistant cell wall, and efficient carotenoid recovery, which collectively contribute to production complexity and cost [4,8]. These limitations have encouraged continued exploration of alternative biological resources for the recovery of carotenoid-rich bioactive extracts. Freshwater macroalgae represent renewable biomass resources containing diverse phytochemicals, although their potential for high-value carotenoid recovery remains comparatively underexplored [9]. Among these, *Chara corallina* is a freshwater macroalga for which previous studies have demonstrated the presence of phenolic, flavonoid, and tannin constituents together with antioxidant activity in extracts obtained using different solvent systems [10]. More recent investigations have also reported antioxidant and anti-tyrosinase activities of *C. corallina* extracts, indicating that this species represents a potentially valuable source of biologically active phytochemicals [11]. However, these previous investigations have largely focused on crude extracts, general phytochemical composition, and associated biological activities rather than targeted recovery of astaxanthin-rich fractions. To the best of our knowledge, no previous study has integrated solvent selection and ultrasound-assisted extraction with HPLC-based confirmation and quantification of astaxanthin and subsequent cellular evaluation of antioxidant, anti-inflammatory, and photoaging-related activities in *C. corallina*-derived extracts. Therefore, *C. corallina* warrants further investigation as an underexplored freshwater source of carotenoid-containing bioactive extracts.

Importantly, the present study does not seek to establish *C. corallina* as quantitatively superior to, or commercially competitive with, established astaxanthin-producing microalgae such as *Haematococcus pluvialis*. Rather, its relevance lies in its status as an underexplored freshwater macroalgal biomass with potential for the recovery of carotenoid-containing bioactive extracts. Previous investigations of freshwater Charophyceae have demonstrated considerable interspecific and environmentally associated variation in carotenoid composition, with carotenoids such as β-carotene, γ-carotene, lutein, neoxanthin, and violaxanthin reported among different *Chara* species [12,13]. However, quantitative information specifically concerning astaxanthin accumulation in *C. corallina* and closely related freshwater macroalgae remains limited, precluding a robust conclusion that its astaxanthin concentration is intrinsically distinct or superior. Accordingly, the significance of *C. corallina* in the present study is based not solely on astaxanthin recovery but also on the biological profile of the resulting extract. Previous studies of *C. corallina* have primarily demonstrated antioxidant and anti-tyrosinase activities of crude extracts [10,11], whereas the present study extends this evidence by evaluating an astaxanthin-containing extract across complementary endpoints related to radical-scavenging activity, antioxidant- and inflammation-associated gene expression, *COL1A2* expression, and inhibition of extracellular matrix-degrading enzymes. This broader biological profile supports further investigation of *C. corallina* as a source of multifunctional carotenoid-containing extracts rather than as a direct replacement for established commercial astaxanthin sources.

Ultrasound-assisted extraction (UAE) has emerged as an efficient and environmentally friendly extraction technology capable of improving the recovery of thermolabile phytochemicals while reducing extraction time, solvent consumption, and energy requirements compared with conventional extraction methods. Acoustic cavitation generated during ultrasonication disrupts cellular structures, enhances solvent penetration, and facilitates mass transfer, thereby improving extraction efficiency while preserving bioactive compounds [14,15]. Although UAE has been successfully applied for the recovery of carotenoids and other phytochemicals from various plant and algal sources, most previous studies have primarily emphasized extraction yield or chemical antioxidant activity, with relatively few investigations demonstrating whether the recovered extracts retain biologically relevant activities associated with skin health [14,16]. Consequently, the relationship between selected extraction conditions, astaxanthin recovery, and multifunctional biological activities remains insufficiently understood.

Therefore, the present study aimed to develop a selected ultrasound-assisted extraction strategy for producing an astaxanthin-rich extract from the freshwater macroalga *Chara corallina* using different solvent systems and to comprehensively evaluate its biological activities relevant to skin health. The selected extract was chemically characterized by high-performance liquid chromatography (HPLC) and subsequently evaluated for antioxidant activity, cytocompatibility, modulation of antioxidant- and inflammation-associated gene expression, regulation of *COL1A2* expression, and inhibition of extracellular matrix-degrading enzymes. By integrating solvent selection under defined UAE conditions with HPLC–PDA-based assessment and biological evaluation, the present study aimed to provide initial information regarding the recovery and biological properties of a carotenoid-containing extract from the underexplored freshwater macroalga *C. corallina*.

## 2. Materials and Methods

### 2.1. Collection and Identification of Chara corallina

*Chara corallina* was collected from a freshwater pond located at Rajamangala University of Technology Srivijaya, Thung Yai Campus, Nakhon Si Thammarat Province, Thailand (8.164° N, 99.676° E), between 08:00 and 10:00 h during the dry season (March 2025). All biomass used in the present study originated from this single sampling site and collection period and was processed as one collected biomass batch for subsequent extraction experiments. Healthy thalli showing no visible signs of epiphytic contamination, discoloration, or mechanical damage were manually harvested from shallow water (approximately 20–50 cm depth). Collected samples were immediately rinsed with pond water to remove loosely attached debris and transported to the laboratory in insulated containers maintained at 4–10 °C for processing within 4 h after collection.

In the laboratory, algal samples were thoroughly washed with running tap water followed by sterile distilled water to remove sediment, epiphytes, and other foreign materials. Species identification was performed based on macroscopic and microscopic morphological characteristics using established taxonomic keys for the genus *Chara*, including thallus architecture, cortical arrangement, stipulodes, branchlets, spine cells, and reproductive structures. Species identification was further confirmed by an algal taxonomist (Dr. Wanninee Chankaew, Faculty of Agriculture, Rajamangala University of Technology Srivijaya, Nakhon Si Thammarat, Thailand). A voucher specimen (Voucher No. CC-2025-001) was deposited in the Herbarium of the Faculty of Veterinary Medicine, Rajamangala University of Technology Srivijaya, Thailand, for future reference.

### 2.2. Preparation of Chara corallina Biomass

Following taxonomic identification, the algal biomass was manually inspected to remove residual foreign materials and damaged tissues. Clean samples were cut into small fragments (approximately 1–2 cm in length) and dried in a forced-air oven at 40 °C until a constant weight was achieved to minimize thermal degradation of carotenoids and other heat-sensitive bioactive compounds. The dried biomass was subsequently ground using a laboratory grinder and passed through a 60-mesh sieve to obtain a homogeneous powder.

The powdered biomass was transferred into airtight, light-resistant containers containing silica gel desiccant and stored at −20 °C until extraction. All sample preparation procedures were performed under reduced light conditions to minimize carotenoid oxidation and preserve the stability of astaxanthin prior to ultrasound-assisted extraction.

### 2.3. Ultrasound-Assisted Extraction (UAE)

Ultrasound-assisted extraction (UAE) was employed to recover astaxanthin-containing extracts from *Chara corallina* biomass. Briefly, 52.5 g of dried algal powder was mixed with the respective extraction solvent at a solid-to-solvent ratio of 1:20 (*w*/*v*) in a sealed amber glass flask. Three solvent systems were evaluated under otherwise identical UAE conditions: ethanol, ethyl acetate, and an ethanol–ethyl acetate mixture containing 48% (*v*/*v*) ethanol. The 48% (*v*/*v*) ethanol composition was selected during preliminary method development as a candidate mixed-solvent system because it provided favorable astaxanthin recovery relative to the other mixed-solvent compositions examined during preliminary testing. This composition was subsequently included, together with ethanol and ethyl acetate as single-solvent comparators, in the main extraction experiment. The preliminary screening was used only to select a candidate mixed-solvent composition and was not intended as a formal optimization study. Among the three solvent systems evaluated in the main experiment, 48% (*v*/*v*) ethanol in ethyl acetate provided the highest astaxanthin recovery and was therefore selected for subsequent biological evaluation.

Extraction was performed using an ultrasonic bath (Elmasonic S 120 H, Elma Schmidbauer GmbH, Singen, Germany) operating at an ultrasonic frequency of 37 kHz and a nominal ultrasonic power of 200 W. Samples were sonicated for 15 min under reduced-light conditions, with the extraction vessels wrapped in aluminum foil to minimize light-induced degradation of carotenoids. During sonication, the bath temperature was monitored throughout the extraction period to minimize heat accumulation and was maintained within 30 ± 2 °C. When necessary, bath water was refreshed solely to maintain this predefined temperature range. The same temperature-control criterion was applied consistently to all extraction treatments.

Following the first extraction, the extracts were centrifuged at 5000× *g* for 10 min at room temperature, and the supernatants were collected. The residual biomass was subsequently re-extracted once with fresh solvent under the same extraction conditions. The supernatants obtained from the two sequential extraction cycles were combined for each independent extraction replicate. The pooled extracts were filtered through Whatman No. 1 filter paper before solvent removal under reduced pressure using a rotary evaporator (Rotavapor R-100, BÜCHI Labortechnik AG, Flawil, Switzerland) at 40 °C.

Each extraction condition was independently prepared and analyzed in triplicate (*n* = 3) using separate aliquots derived from the same collected biomass batch. These replicates therefore represent independent extraction replicates rather than independent field-level biological replicates.

### 2.4. HPLC–PDA Analysis and Quantification of the Astaxanthin-Corresponding Component

The *C. corallina* extracts were analyzed by HPLC–PDA to evaluate a chromatographic component corresponding to an authentic astaxanthin reference standard and to estimate its concentration by external calibration. Chromatographic correspondence was assessed based on retention-time agreement and UV–visible spectral characteristics relative to the authentic reference standard. Because orthogonal structural analysis such as LC–MS/MS was not performed, the chromatographic component was conservatively interpreted as an astaxanthin-corresponding component rather than as definitive structural identification of a specific molecular form. Prior to analysis, each dried extract was dissolved in HPLC-grade methanol (Merck KGaA, Darmstadt, Germany), sonicated for 5 min to ensure complete dissolution, and filtered through a 0.22 μm PTFE syringe filter into amber HPLC vials.

Chromatographic analysis was performed using an Agilent 1260 HPLC system (Agilent Technologies, Santa Clara, CA, USA) equipped with a photodiode array (PDA) detector. Separation was achieved on a C30 reversed-phase column (250 × 4.6 mm, 5 μm; YMC Co., Kyoto, Japan) maintained at 30 °C. The mobile phase consisted of methanol (A) and acetonitrile (B), and separation was performed using the following gradient program: 0–5 min, 70% A and 30% B; 5–15 min, a linear gradient from 70% A/30% B to 50% A/50% B; 15–25 min, a linear gradient to 20% A/80% B; 25–30 min, 20% A/80% B; 30–32 min, a return to the initial composition of 70% A/30% B; followed by re-equilibration at 70% A/30% B until 37 min. The flow rate was 1.0 mL min^−1^, the injection volume was 20 μL, and chromatograms were monitored at 476 nm. The total run time, including column re-equilibration, was 37 min [17,18].

Astaxanthin was assigned based on comparison of the retention time and UV–visible spectral characteristics of the sample peak with those of an authentic astaxanthin reference standard (≥98% purity; Sigma-Aldrich, St. Louis, MO, USA). Quantification was performed using an external calibration curve generated from the authentic standard. The HPLC–PDA method employed in the present study was not specifically designed or validated to resolve free astaxanthin from esterified derivatives or to distinguish individual *E*/*Z* geometric isomers. Therefore, the quantified chromatographic component was not assigned to a specific molecular form of astaxanthin and is reported conservatively as an astaxanthin-corresponding component based on chromatographic and UV–visible spectral correspondence with the authentic standard.

The selected CCE was evaluated in subsequent biological assays as a chemically complex algal extract. Purified astaxanthin was not included as a biological comparator; therefore, these assays were designed to characterize the biological responses associated with CCE rather than to determine the specific contribution or relative potency of astaxanthin.

### 2.5. DPPH Radical Scavenging Assay

The radical-scavenging activity of CCE was evaluated using the 2,2-diphenyl-1-picrylhydrazyl (DPPH) radical scavenging assay with minor modifications to a previously described method [19]. Dried extracts were dissolved in methanol (RCI Labscan Limited, Bangkok, Thailand) and serially diluted to final concentrations of 1.25–40 μg mL^−1^.

Briefly, 100 μL of each extract solution was mixed with an equal volume of freshly prepared 0.2 mM DPPH (Sigma-Aldrich, St. Louis, MO, USA) solution in a 96-well microplate. The reaction mixtures were incubated at room temperature in the dark for 30 min to minimize light-induced degradation of both DPPH and carotenoids. Absorbance was then measured at 517 nm using a BioTek Synergy HTX Multi-Mode Microplate Reader (Agilent Technologies, Santa Clara, CA, USA).

Methanol containing DPPH without extract served as the negative control, whereas Trolox (Sigma-Aldrich, St. Louis, MO, USA) was used as the reference antioxidant and was assayed under the same experimental conditions as the CCE samples. Sample blanks containing the extract solution without DPPH were included to correct for background absorbance resulting from the natural color of the extracts. Radical scavenging activity was determined from the reduction in absorbance relative to the negative control. The half-maximal inhibitory concentration (IC_50_) was calculated by nonlinear regression analysis using GraphPad Prism version 11.0.2 (GraphPad Software, Boston, MA, USA), with lower IC_50_ values indicating greater antioxidant activity. All measurements were performed using three independently prepared extract samples, each analyzed in triplicate.

### 2.6. Cell Culture

Human dermal fibroblasts (HDFs; ATCC PCS-201-012) and murine macrophage RAW264.7 cells (ATCC TIB-71) were obtained from the American Type Culture Collection (ATCC, Manassas, VA, USA). HDF cells were used as an in vitro model to evaluate antioxidant- and photoaging-related cellular responses because they are the principal cells responsible for extracellular matrix synthesis and collagen homeostasis in the dermis, whereas RAW264.7 macrophages were used to investigate the anti-inflammatory activity of the extracts under lipopolysaccharide (LPS)-induced inflammatory conditions [20,21].

HDF cells were cultured in Dulbecco’s Modified Eagle Medium (DMEM, low glucose) supplemented with 10% (*v*/*v*) heat-inactivated fetal bovine serum (FBS), 100 U mL^−1^ penicillin, and 100 μg mL^−1^ streptomycin. RAW264.7 cells were maintained in RPMI-1640 medium containing the same supplements. All culture media and supplements were obtained from Gibco (Thermo Fisher Scientific, Waltham, MA, USA). Both cell lines were incubated at 37 °C in a humidified atmosphere of 5% CO_2_, and the culture medium was replaced every 2–3 days.

Cells were routinely subcultured at approximately 80–90% confluence. HDF cells were detached using 0.25% trypsin–0.02% EDTA (Gibco, Thermo Fisher Scientific, Waltham, MA, USA), whereas RAW264.7 cells were harvested using a sterile cell scraper to preserve membrane integrity. Cell number and viability were determined by the trypan blue exclusion assay using a hemocytometer, and only cell suspensions with >95% viability were used for subsequent experiments.

HDF cells between passages 5 and 20 were used throughout the study. This passage range was predefined to provide sufficient cell availability across independent experiments while avoiding extensively passaged cultures. Cells were routinely examined for the expected fibroblast-like morphology before experimental use. Because passage-dependent phenotypic and transcriptional changes may occur in dermal fibroblasts, the relatively broad passage range employed in the present study is recognized as a methodological limitation.

Before treatment, cells were allowed to attach for 24 h under standard culture conditions. Unless otherwise specified, the astaxanthin-rich extract was dissolved in dimethyl sulfoxide (DMSO; Merck KGaA, Darmstadt, Germany), sterilized through a 0.22 μm membrane filter, and diluted with culture medium immediately before use. The final DMSO concentration in all treatment groups did not exceed 0.1% (*v*/*v*), and the same concentration of DMSO was included in the vehicle control.

### 2.7. Cell Viability Assay

The cytocompatibility of the astaxanthin-rich *Chara corallina* extract was evaluated using the 3-(4,5-dimethylthiazol-2-yl)-2,5-diphenyltetrazolium bromide (MTT; Sigma-Aldrich, St. Louis, MO, USA) assay with minor modifications to a previously described method [22]. HDF and RAW264.7 cells were seeded into 96-well plates at a density of 1 × 10^4^ cells per well and allowed to attach for 24 h under the culture conditions described in Section 2.6.

For HDF cells, CCE was evaluated at 0 (vehicle control), 12.5, 25, 50, and 100 μg mL^−1^. For RAW264.7 cells, CCE was evaluated at 0 (vehicle control), 12.5, 25, and 50 μg mL^−1^. Cell viability was assessed after 24 h of exposure using the MTT assay. Concentrations that maintained acceptable cell viability were subsequently selected for the corresponding cellular experiments.

Following treatment, the culture medium was removed and replaced with 100 μL of MTT solution (0.5 mg mL^−1^ prepared in serum-free medium). After incubation at 37 °C for 4 h, the resulting intracellular formazan crystals were dissolved in 100 μL DMSO with gentle shaking for 10 min at room temperature. Absorbance was measured at 570 nm using a BioTek Synergy HTX Multi-Mode Microplate Reader (Agilent Technologies, Santa Clara, CA, USA). When applicable, absorbance at 630 nm was used as the reference wavelength for background correction.

Cell viability was expressed as a percentage relative to the vehicle control, which was defined as 100% viability. Extract concentrations maintaining ≥90% cell viability were considered non-cytotoxic and were subsequently selected for gene expression and anti-photoaging enzyme inhibition assays. All experiments were performed using three independent biological replicates, each analyzed with three technical replicates.

### 2.8. UV-Induced Photoaging Model in Human Dermal Fibroblasts

An in vitro UV-induced photoaging model was established using human dermal fibroblasts (HDFs) to evaluate the effects of the astaxanthin-rich *Chara corallina* extract on antioxidant- and extracellular matrix-related gene expression. HDF cells were seeded into appropriate culture plates according to the requirements of each downstream assay and cultured under the conditions described in Section 2.6. After 24 h of attachment, the culture medium was replaced with serum-free medium, and the cells were serum-starved overnight before UV irradiation [23,24].

Immediately before irradiation, the culture medium was removed, and the cells were washed twice with sterile phosphate-buffered saline (PBS). A thin layer of PBS was retained to prevent cellular dehydration during UV exposure. HDF cells were sequentially exposed to UVA (λmax = 365 nm; 3 J cm^−2^) followed by UVB (λmax = 316 nm; 300 mJ cm^−2^) using 18 W Philips UV lamps (Philips, Eindhoven, The Netherlands) according to a previously published protocol with minor modifications [23,24]. Throughout irradiation, the distance between the UV source and the cell monolayer was kept constant to ensure experimental reproducibility. The selected UVA and UVB doses were chosen based on a previously validated in vitro photoaging model that induces measurable oxidative stress and photoaging-associated molecular responses while maintaining acceptable cell viability [23,24].

Immediately after irradiation, PBS was replaced with fresh culture medium containing the astaxanthin-rich extract at final concentrations of 12.5, 25, or 50 μg mL^−1^. Cells were further incubated for 24 h before gene expression analysis. The experimental groups consisted of a UV-irradiated vehicle control and UV-irradiated cells treated with CCE at 12.5, 25, or 50 μg mL^−1^. The UV-irradiated vehicle control was used as the calibrator for relative gene-expression analysis. No nonirradiated normal control or positive-control treatment was included in this experiment. 

Unless otherwise specified, the final DMSO concentration did not exceed 0.1% (*v*/*v*) in any treatment group. All experiments were independently repeated three times.

### 2.9. LPS-Induced Inflammatory Model in RAW264.7 Macrophages

An LPS-induced inflammatory model was established in RAW264.7 murine macrophages to evaluate the effects of the astaxanthin-rich *Chara corallina* extract on inflammation-associated gene expression. RAW264.7 cells were seeded into appropriate culture plates according to the requirements of each downstream assay and cultured under the conditions described in Section 2.6. After 24 h of attachment, the culture medium was replaced with fresh complete medium containing 1 μg mL^−1^ lipopolysaccharide (LPS; *Escherichia coli* O111:B4; Sigma–Aldrich, St. Louis, MO, USA) to induce an inflammatory response [25,26].

RAW264.7 cells were assigned to four experimental groups: an LPS-stimulated vehicle control and LPS-stimulated cells treated with CCE at final concentrations of 12.5, 25, or 50 μg mL^−1^. Cells were incubated for an additional 24 h under standard culture conditions. The LPS-stimulated vehicle control was used as the calibrator for evaluating CCE-associated changes in inflammation-related gene expression. No unstimulated normal control or positive-control treatment was included in this experiment. 

Unless otherwise specified, the final DMSO concentration did not exceed 0.1% (*v*/*v*) in any treatment group. Following treatment, total RNA was isolated for subsequent gene expression analysis as described in Section 2.10. All experiments were independently repeated three times.

### 2.10. Gene Expression Analysis by One-Step RT-qPCR

Total RNA was isolated from treated and control cells using the FavorPrep™ Tissue Total RNA Extraction Mini Kit (Favorgen Biotech Corp., Ping-Tung, Taiwan) according to the manufacturer’s instructions. Briefly, the culture medium was removed, the cells were washed twice with ice-cold phosphate-buffered saline (PBS), and cell lysis was performed directly in the culture plates using the supplied lysis buffer. Total RNA was purified using silica membrane spin columns, eluted in 30–50 μL of RNase-free water, and stored at −80 °C until analysis.

RNA concentration and purity were determined using a NanoDrop Lite Spectrophotometer (Thermo Fisher Scientific, Waltham, MA, USA). Only RNA samples with an A_260_/A_280_ ratio of 1.8–2.1 were used for subsequent analysis. Before one-step reverse transcription quantitative PCR (RT-qPCR), RNA samples were diluted with RNase-free water to a working concentration of 1 ng μL^−1^.

One-step RT-qPCR was performed using the SensiFAST™ SYBR^®^ No-ROX One-Step Kit (Bioline Reagents Ltd., London, UK; Meridian Bioscience, Cincinnati, OH, USA) on a CFX96 Real-Time PCR Detection System (Bio-Rad Laboratories, Hercules, CA, USA). Each 20 μL reaction contained 10 μL of 2× SensiFAST SYBR No-ROX One-Step Mix, 0.8 μL each of forward and reverse primers (10 μM), 0.2 μL of reverse transcriptase, 0.4 μL of RiboSafe RNase Inhibitor, 4 μL of RNA template at 1 ng μL^−1^, and 3.8 μL of nuclease-free water.

The thermal cycling program consisted of reverse transcription at 45 °C for 10 min and polymerase activation at 95 °C for 2 min, followed by 40 cycles of denaturation at 95 °C for 5 s and annealing/extension at 60 °C for 20 s. Fluorescence was recorded at the end of each annealing/extension step. A post-amplification melting-curve analysis was performed to confirm reaction specificity, and only reactions producing a single melting peak were included in the analysis.

Primer sequences, target genes, amplicon sizes, annealing temperatures, and amplification efficiencies are provided in Table S1. Amplification efficiency was assessed using standard curves, and primer pairs with efficiencies of 90–110% and coefficients of determination of R^2^ ≥ 0.98 were considered acceptable. Each RT-qPCR run included a no-template control and a no-reverse-transcription control to assess reagent contamination and genomic DNA amplification, respectively.

Target-gene expression was normalized to glyceraldehyde-3-phosphate dehydrogenase (*GAPDH*) as the reference gene. Relative expression was calculated using the comparative 2^−ΔΔCt^ method [27]. RT-qPCR analyses were conducted in three independently performed experiments (*n* = 3). For each independent experiment, qPCR reactions were performed in technical triplicate. The technical Ct values were averaged before relative gene-expression calculations, and the resulting expression value from each independent experiment was treated as a single experimental unit for statistical analysis. Thus, *n* = 3 represents independent experiments and not individual qPCR wells. RNA handling, assay validation, and reporting were performed in accordance with the Minimum Information for Publication of Quantitative Real-Time PCR Experiments (MIQE) guidelines [28].

### 2.11. Antioxidant and Extracellular Matrix-Related Gene Expression Analysis

The effects of the selected *Chara corallina* extract (CCE) on antioxidant- and extracellular matrix-related gene expression were evaluated in UV-irradiated human dermal fibroblasts (HDFs) using the experimental conditions described in Section 2.8. HDFs were seeded into 24-well culture plates at a density of 4 × 10^5^ cells per well and assigned to four experimental groups: a UV-irradiated vehicle control and UV-irradiated cells treated with CCE at final concentrations of 12.5, 25, or 50 μg mL^−1^. All experimental groups were subjected to the same UV-irradiation protocol. The UV-irradiated vehicle-control group received the corresponding vehicle without CCE, whereas the treatment groups received CCE at the indicated concentrations.

Following a 24 h treatment period, total RNA was isolated and analyzed by one-step RT-qPCR according to the procedure described in Section 2.10. The expression levels of three antioxidant-related genes (*SOD1*, *GPX1*, and *CAT*) and one extracellular matrix-related gene (*COL1A2*) were determined. Relative gene expression was normalized to *GAPDH* and calculated using the 2^−ΔΔCt^ method [27], with the UV-irradiated vehicle control used as the calibrator (relative expression = 1). Accordingly, the analysis was designed to evaluate CCE-associated changes in gene expression relative to UV-irradiated vehicle-treated cells and was not intended to quantify UV-induced transcriptional changes relative to a nonirradiated baseline.

RT-qPCR analyses were conducted in three independently performed experiments (*n* = 3). For each independent experiment, qPCR reactions were performed in technical triplicate. The technical Ct values were averaged before relative gene-expression calculations, and the resulting expression value from each independent experiment was treated as a single experimental unit for statistical analysis. Thus, *n* = 3 represents independent experiments and not individual qPCR wells.

### 2.12. Anti-Inflammatory Gene Expression Analysis

The effects of CCE on inflammation-related gene expression were evaluated using the LPS-induced inflammatory model described in Section 2.9. RAW264.7 macrophages were seeded into 24-well culture plates at a density of 1.5 × 10^6^ cells per well and stimulated with 1 μg mL^−1^ lipopolysaccharide (LPS) followed by treatment with CCE at final concentrations of 12.5, 25, or 50 μg mL^−1^.

RAW264.7 macrophages were assigned to four experimental groups: an LPS-stimulated vehicle control and LPS-stimulated cells treated with CCE at final concentrations of 12.5, 25, or 50 μg mL−1. Relative expression of *Nos2*, *Ptgs2*, and *Il1b* was determined using the 2^−ΔΔCt^ method [27]. The LPS-stimulated vehicle control was used as the calibrator (relative expression = 1) for evaluating treatment-associated changes relative to the induced inflammatory state. Accordingly, the analysis was designed to evaluate CCE-associated changes in inflammation-related gene expression relative to LPS-stimulated vehicle-treated cells and was not intended to quantify LPS-induced transcriptional changes relative to an unstimulated baseline.

Following a 24 h treatment period, total RNA was isolated and analyzed by one-step RT-qPCR according to the procedure described in Section 2.10. The expression of three inflammation-associated genes (*Nos2*, *Ptgs2*, and *Il1b*) was determined. Relative gene expression was normalized to *Gapdh*. RT-qPCR analyses were conducted in three independently performed experiments (*n* = 3). For each independent experiment, qPCR reactions were performed in technical triplicate. The technical Ct values were averaged before relative gene-expression calculations, and the resulting expression value from each independent experiment was treated as a single experimental unit for statistical analysis. Thus, *n* = 3 represents independent experiments and not individual qPCR wells.

### 2.13. Anti-Photoaging Enzyme Inhibition Assays

The anti-photoaging potential of the astaxanthin-rich *Chara corallina* extract was evaluated by determining its inhibitory activity against three extracellular matrix-degrading enzymes, namely collagenase, elastase, and hyaluronidase, using commercially available colorimetric assay kits (Elabscience Biotechnology Inc., Wuhan, China). These enzymes were selected because of their established roles in collagen degradation, elastin breakdown, and extracellular matrix remodeling during skin photoaging [29,30].

The extract was dissolved in dimethyl sulfoxide (DMSO) to prepare a 1 mg mL^−1^ stock solution and diluted with the corresponding assay buffer to final concentrations of 12.5, 25, and 50 μg mL^−1^. The final DMSO concentration in all reaction mixtures was maintained below 1% (*v*/*v*). All assays were performed according to the manufacturer’s instructions with minor modifications. Blank reactions containing assay buffer without enzyme were included for background correction, whereas enzyme solutions without extract served as the negative controls. Epigallocatechin gallate (EGCG; Sigma-Aldrich, St. Louis, MO, USA) at 20 μM was used as the positive inhibitory control in the collagenase, elastase, and hyaluronidase inhibition assays.

For the collagenase inhibition assay, the extract was incubated with collagenase and the corresponding chromogenic substrate at 37 °C for 30 min, and absorbance was measured at 345 nm using a BioTek Synergy HTX Multi-Mode Microplate Reader (Agilent Technologies, Santa Clara, CA, USA). Elastase inhibitory activity was evaluated by incubating the extract with porcine pancreatic elastase and the supplied chromogenic substrate at 37 °C for 20 min, followed by absorbance measurement at 410 nm. Hyaluronidase inhibition was determined by incubating the extract with hyaluronidase at 37 °C for 30 min prior to substrate addition according to the manufacturer’s protocol. The reaction was terminated using the supplied stop solution, and absorbance was recorded at 450 nm.

Enzyme-inhibitory activity was calculated as percentage inhibition relative to the untreated enzyme control after correction for the corresponding sample blank, according to the manufacturer’s instructions. Because 50% inhibition was not reached within the experimentally evaluated concentration range for all three enzymes, IC_50_ values were not estimated by extrapolation beyond the observed data. Accordingly, enzyme-inhibitory activity was reported as the experimentally observed percentage inhibition at each tested CCE concentration. Each assay was independently performed three times (*n* = 3 independent experiments), with three technical replicate measurements within each independent experiment. Technical replicate measurements were averaged to obtain a single value for each condition within each independent experiment and were not treated as independent observations for statistical analysis.

### 2.14. Statistical Analysis

All experiments were performed using at least three independent biological replicates, and the results are presented as mean ± standard deviation (SD). Prior to statistical analysis, data were assessed for normality using the Shapiro–Wilk test and for homogeneity of variances using Levene’s test [31,32]. Data satisfying both assumptions were analyzed using parametric statistical methods.

All cellular experiments were independently performed three times (*n* = 3 independent experiments). Within each independent experiment, measurements were performed in three technical replicate wells. Technical replicates represent repeated measurements of the same experimental condition within a single independent experiment and were used to account for within-assay variability; they were not considered independent experimental units. The three technical replicate values were averaged to obtain a single value for each condition within each independent experiment. Consequently, only the three independent experiment-level values (*n* = 3) were used for inferential statistical analyses.

Comparisons among multiple experimental groups were performed using one-way analysis of variance (ANOVA) followed by Tukey’s multiple comparison test. When only two groups were compared, an unpaired two-tailed Student’s *t*-test was applied, as appropriate.

For the DPPH radical-scavenging assay, dose–response curves were generated by nonlinear regression, and the half-maximal inhibitory concentration (IC50) was calculated using GraphPad Prism version 11.0.2 (GraphPad Software Inc., San Diego, CA, USA). For the extracellular matrix-degrading enzyme inhibition assays, inhibitory activity was reported as the experimentally observed percentage inhibition at each tested CCE concentration because 50% inhibition was not reached within the evaluated concentration range. Relative gene-expression data were analyzed using fold-change values obtained by the comparative 2^−ΔΔCt^ method as described in Section 2.10 [27].

All statistical analyses were performed using GraphPad Prism version 11.0.2 (GraphPad Software Inc., San Diego, CA, USA). Differences were considered statistically significant at *p* < 0.05.

## 3. Results

### 3.1. Preparation of the Selected CCE and HPLC Characterization

**Figure 1:**
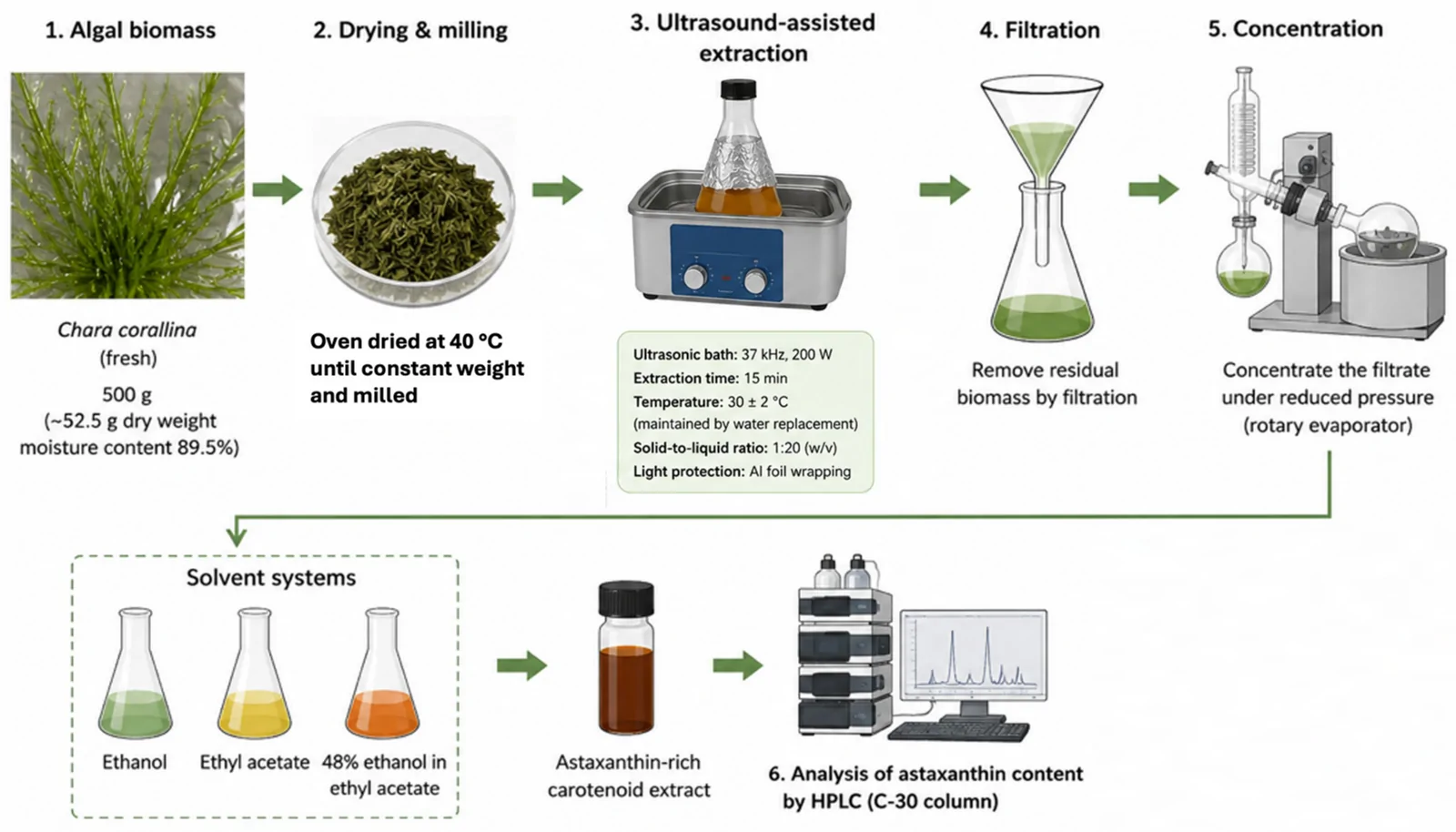
Schematic workflow of ultrasound-assisted extraction of an astaxanthin-rich carotenoid extract from *Chara corallina*. Fresh algal biomass was extracted with ethanol, ethyl acetate, or 48% ethanol in ethyl acetate at a solvent-to-biomass ratio of 20:1 (*v*/*w*), 30 °C, and 200 W for 15 min. The extracts were subsequently separated, concentrated under reduced pressure, and analyzed for astaxanthin content using HPLC.

The overall workflow for preparing the astaxanthin-rich extract from *Chara corallina* is illustrated in Figure 1. Three solvent systems, including ethanol, ethyl acetate, and a selected mixed-solvent system consisting of 48% ethanol in ethyl acetate, were evaluated under identical ultrasound-assisted extraction conditions to determine the solvent system providing the highest astaxanthin recovery.

The extraction solvent markedly influenced astaxanthin yield (Table 1). Ethanol produced the lowest yield (45.8 ± 2.1 mg), corresponding to 0.0873 ± 0.0042% (*w*/*w*) of dry biomass. Extraction with ethyl acetate increased the yield to 102.5 ± 3.7 mg (0.1952 ± 0.0071% *w*/*w*). Among the evaluated solvent systems, the selected mixed-solvent system (48% ethanol in ethyl acetate) produced the highest astaxanthin yield of 136.4 ± 4.5 mg, corresponding to 0.2598 ± 0.0086% (*w*/*w*) of dry biomass. This represented approximately a 2.98-fold increase compared with ethanol and a 1.33-fold increase compared with ethyl acetate.

**Figure 2:**
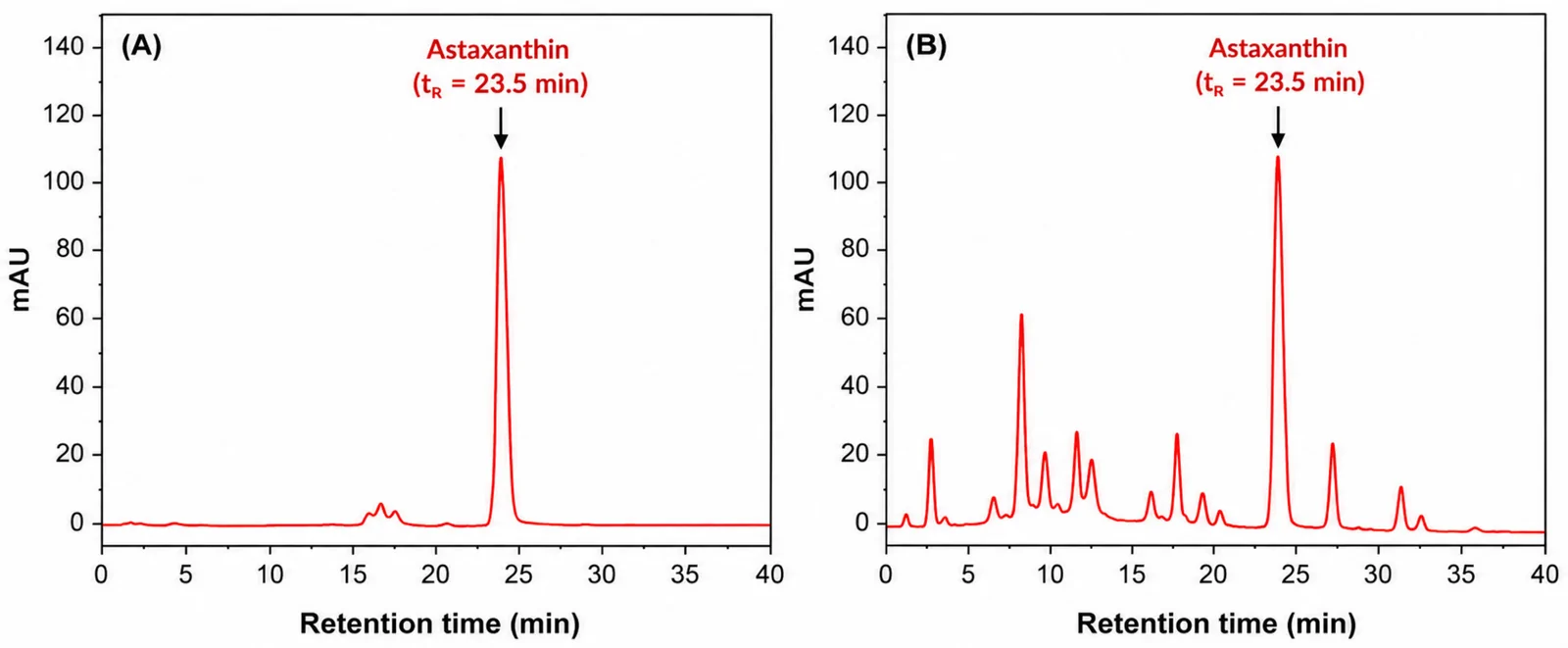
Representative HPLC–PDA chromatograms of (**A**) the authentic astaxanthin reference standard and (**B**) the selected *Chara corallina* extract (CCE). A chromatographic component in CCE showed retention-time and UV–visible spectral characteristics corresponding to those of the authentic astaxanthin reference standard. Additional chromatographic components were observed but were not structurally characterized; therefore, their identities cannot be assigned from the present HPLC–PDA analysis.

HPLC–PDA analysis of CCE revealed a chromatographic component with retention time and UV–visible spectral characteristics corresponding to those of the authentic astaxanthin reference standard (Figure 2). Several additional chromatographic peaks were also observed in the extract. Because these components were not subjected to orthogonal structural characterization, their identities remain unresolved and they cannot be assigned to other carotenoids, astaxanthin derivatives, or potential degradation or oxidation products based on the present HPLC–PDA data alone. Accordingly, the chromatographic results provide evidence of correspondence with the astaxanthin reference standard but do not establish the complete carotenoid composition or chemical integrity of astaxanthin in CCE.

Based on its superior astaxanthin recovery, the extract obtained using the selected solvent system was selected for all subsequent antioxidant, anti-inflammatory, and anti-photoaging evaluations.

### 3.2. Antioxidant Activity

The antioxidant activity of the selected CCE obtained from *Chara corallina* was evaluated using complementary chemical and molecular approaches. Antioxidant capacity was first determined by the DPPH radical scavenging assay, followed by analysis of antioxidant-related gene expression in UV-irradiated human dermal fibroblasts.

#### 3.2.1. DPPH Radical Scavenging Activity

The selected CCE exhibited DPPH radical-scavenging activity with an IC_50_ value of 12.3 ± 0.9 μg/mL, whereas Trolox, used as the reference antioxidant, exhibited an IC_50_ value of 7.3 ± 0.8 μg/mL (Table 2). These results demonstrate the radical-scavenging activity of CCE under the conditions of the DPPH assay.

#### 3.2.2. Upregulation of Antioxidant-Related Genes

**Figure 3:**
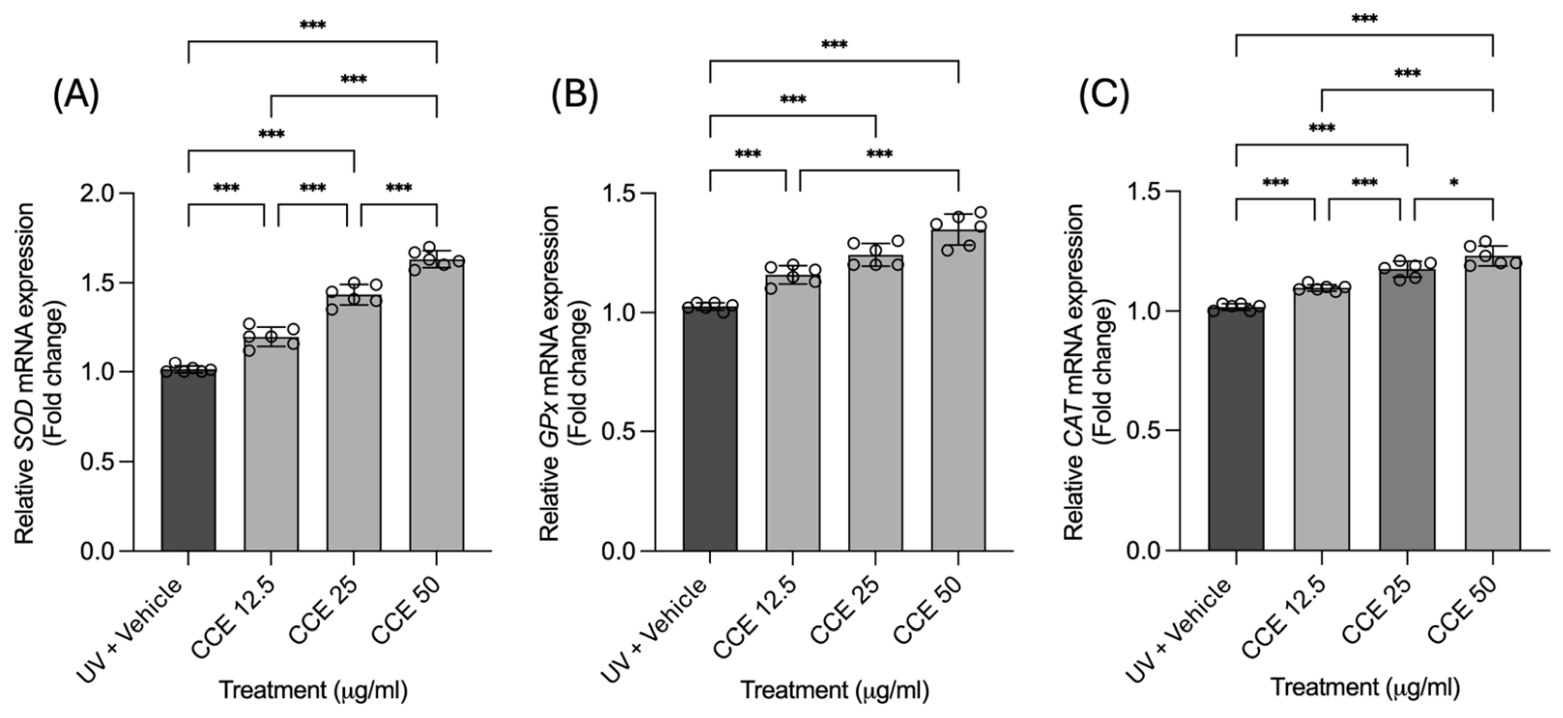
Effects of CCE on antioxidant-related gene expression in UV-irradiated human dermal fibroblasts (HDFs). Relative mRNA expression levels of (**A**) SOD1, (**B**) CAT, and (**C**) GPX1 were determined by RT-qPCR in UV-irradiated cells treated with vehicle or CCE at 12.5, 25, or 50 μg/mL. The UV-irradiated vehicle control (UV + Vehicle) was used as the calibrator for relative gene-expression analysis. Data are presented as mean ± SD from three independent experiments (*n* = 3), with white circles represent individual technical replicate measurements. Technical replicate measurements within each independent experiment were averaged before statistical analysis. Statistical significance between the indicated groups is denoted as *p* < 0.05 (*), and *p* < 0.001 (***), as determined by one-way ANOVA followed by Tukey’s multiple comparison test.

In UV-irradiated HDF cells, treatment with CCE significantly modulated the mRNA expression of the antioxidant-related genes SOD1, CAT, and GPX1 compared with the UV-irradiated vehicle control (Figure 3). These findings demonstrate CCE-associated transcriptional responses under UV-irradiated conditions. However, because a nonirradiated control was not included in this experiment, the magnitude of UV-induced transcriptional changes relative to the nonirradiated baseline could not be determined.

Among the analyzed genes, *SOD1* exhibited the greatest transcriptional response, with relative expression increasing from 1.19 ± 0.05 at 12.5 μg mL^−1^ to 1.63 ± 0.04 at 50 μg mL^−1^. Similarly, *GPX1* expression increased from 1.15 ± 0.03 to 1.34 ± 0.06, whereas *CAT* expression increased from 1.09 ± 0.01 to 1.23 ± 0.04 over the same concentration range. All treatment groups exhibited significantly higher expression levels than the UV-irradiated control (*p* < 0.05).

These findings demonstrate that the selected CCE enhanced the transcription of key antioxidant-related genes associated with cellular antioxidant defense.

### 3.3. Cytocompatibility of the Selected CCE

**Figure 4:**
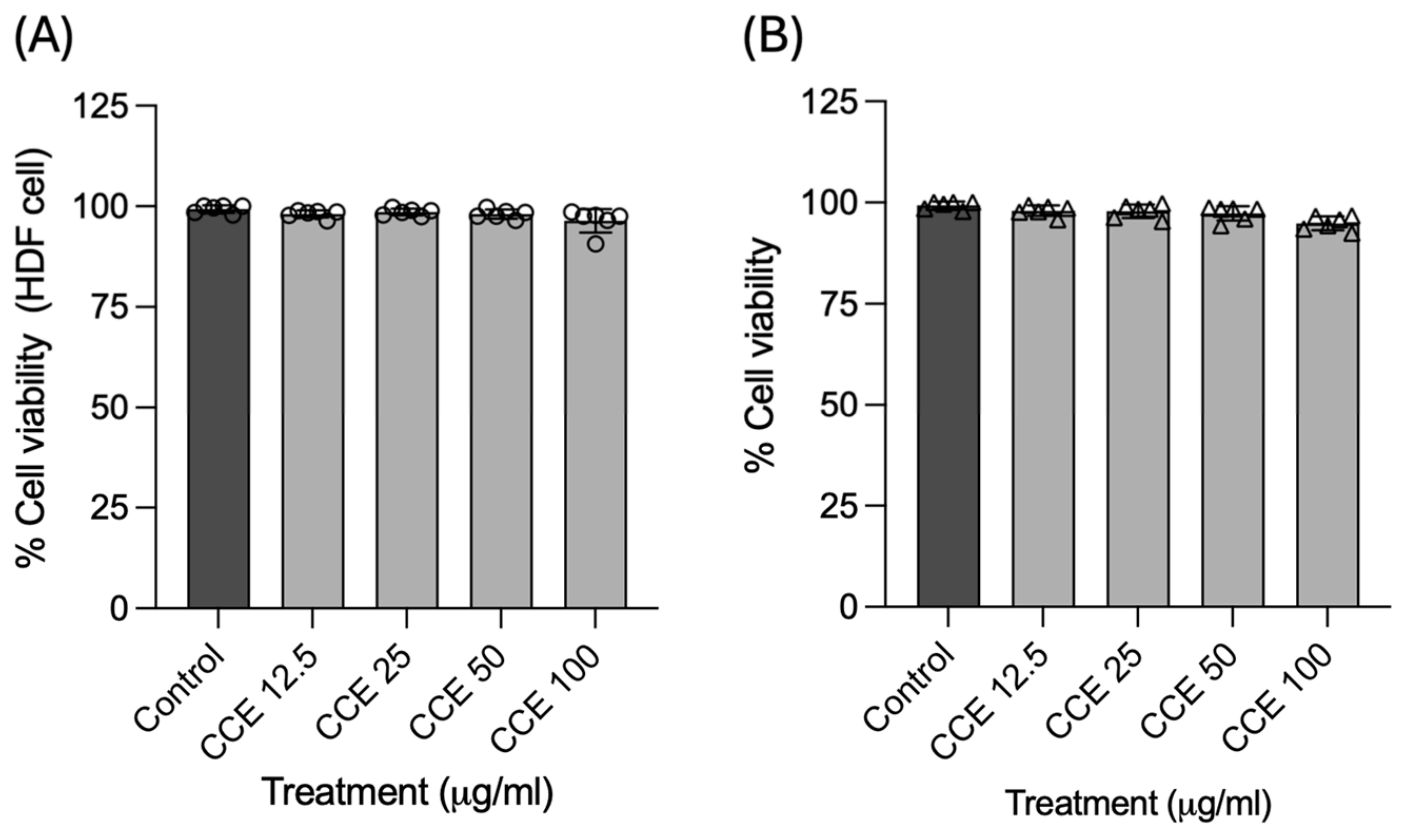
Cytocompatibility of the selected astaxanthin-rich *Chara corallina* extract determined by the MTT assay. (**A**) Viability of human dermal fibroblasts (HDFs) following 24 h treatment with 12.5–100 μg mL^−1^ of the extract. (**B**) Viability of RAW264.7 macrophages following 24 h treatment with 12.5–100 μg mL^−1^ of the extract. Data are presented as mean ± SD from three independent experiments (*n* = 3). White circles represent individual technical replicate measurements for the CCE treatments, whereas white triangles represent individual technical replicate measurements for the EGCG positive control. Technical replicate measurements within each independent experiment were averaged before statistical analysis.

The cytocompatibility of the selected CCE was evaluated in human dermal fibroblasts (HDFs) and RAW264.7 macrophages using the MTT assay. As shown in Figure 4, the extract exhibited high cytocompatibility in both cell lines throughout the tested concentration range.

In HDF cells (Figure 4A), treatment with 12.5–100 μg mL^−1^ of the extract for 24 h maintained cell viability between 96.44 ± 2.91% and 99.33 ± 0.94%. No significant reduction in cell viability was observed at any tested concentration (*p* > 0.05).

Similarly, RAW264.7 macrophages (Figure 4B) maintained high viability following treatment with the selected extract. Cell viability remained between 97.94 ± 1.19% and 98.82 ± 0.97% across the concentrations used for subsequent anti-inflammatory evaluation, with no significant differences compared with the untreated control (*p* > 0.05).

Based on these findings, extract concentrations of 12.5, 25, and 50 μg mL^−1^ were selected for all subsequent cell-based assays.

### 3.4. Anti-Inflammatory Activity

**Figure 5:**
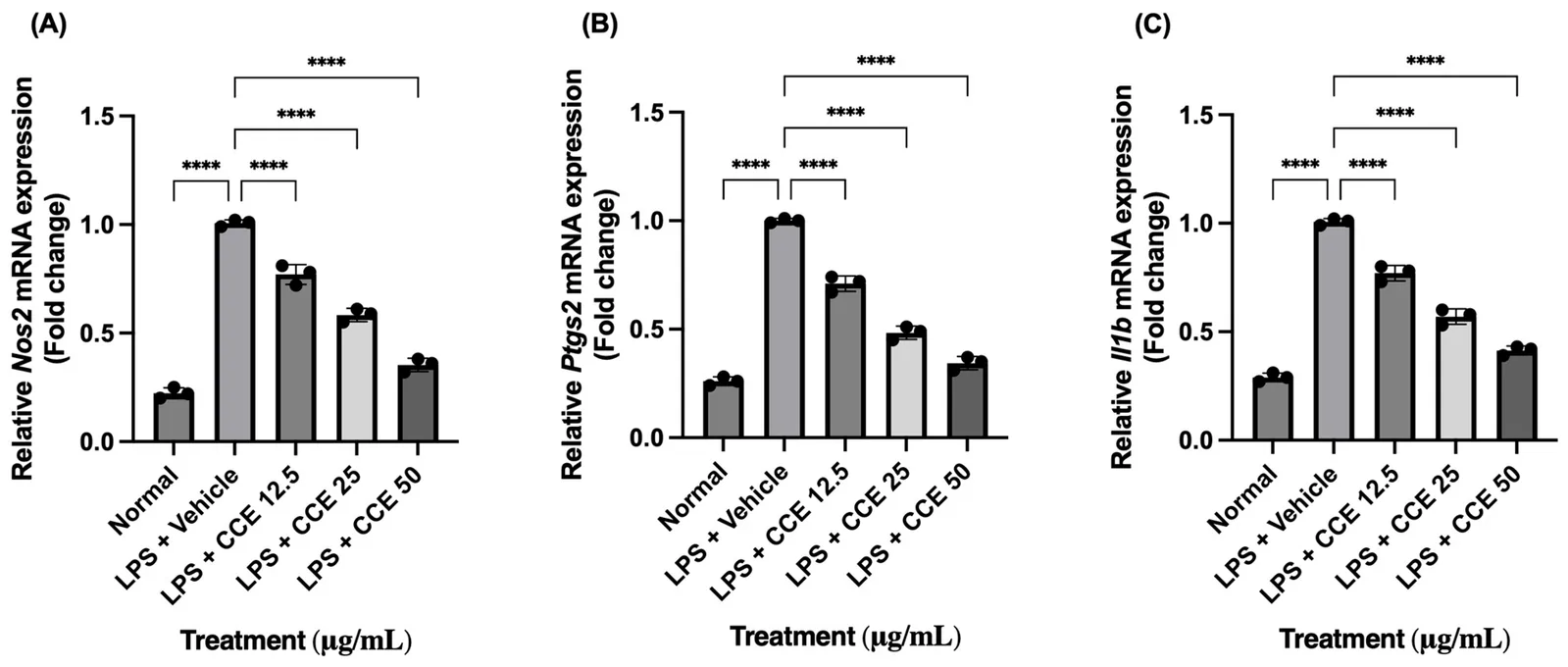
Effects of CCE on LPS-induced inflammation-associated gene expression in RAW264.7 cells. Relative mRNA expression of (**A**) *Nos2*, (**B**) *Ptgs2*, and (**C**) *Il1b* was determined by RT-qPCR. The LPS-stimulated vehicle control was used as the calibrator (relative expression = 1) for evaluating treatment effects relative to the induced inflammatory state. Data are presented as mean ± SD from three independent experiments (*n* = 3). Circles represent individual data points. Technical qPCR replicates were averaged within each independent experiment before statistical analysis. Statistical significance between the indicated groups was analyzed by one-way ANOVA followed by Tukey’s multiple comparison test. *p* < 0.0001 (****).

The anti-inflammatory activity of the selected astaxanthin-rich *Chara corallina* extract was evaluated by determining the expression of inflammation-associated genes in LPS-stimulated RAW264.7 macrophages. CCE treatment reduced the expression of *Nos2*, *Ptgs2*, and *Il1b* relative to the LPS-stimulated vehicle control (Figure 5). 

The expression of *Nos2* decreased from 1.02 ± 0.03 in the LPS-treated control to 0.76 ± 0.08, 0.57 ± 0.04, and 0.34 ± 0.05 following treatment with 12.5, 25, and 50 μg mL^−1^, respectively (Figure 5A). Similarly, *Ptgs2* expression was significantly reduced from 1.01 ± 0.02 in the LPS-treated control to 0.70 ± 0.06, 0.49 ± 0.07, and 0.33 ± 0.04 after treatment with the same concentrations of the extract (Figure 5B). A comparable trend was observed for *Il1b*, with relative expression decreasing from 1.01 ± 0.02 to 0.75 ± 0.08, 0.56 ± 0.05, and 0.41 ± 0.03, respectively (Figure 5C). All treatment groups differed significantly from the LPS-treated control (*p* < 0.05).

Collectively, these findings demonstrate that the selected CCE significantly downregulated the expression of key inflammation-associated genes in LPS-stimulated RAW264.7 macrophages.

### 3.5. Anti-Photoaging Activity

The extracellular matrix-related activity of the selected CCE was evaluated by determining its effects on *COL1A2* mRNA expression in UV-irradiated human dermal fibroblasts and its inhibitory activity against extracellular matrix-degrading enzymes.

#### 3.5.1. Upregulation of COL1A2 Expression

**Figure 6:**
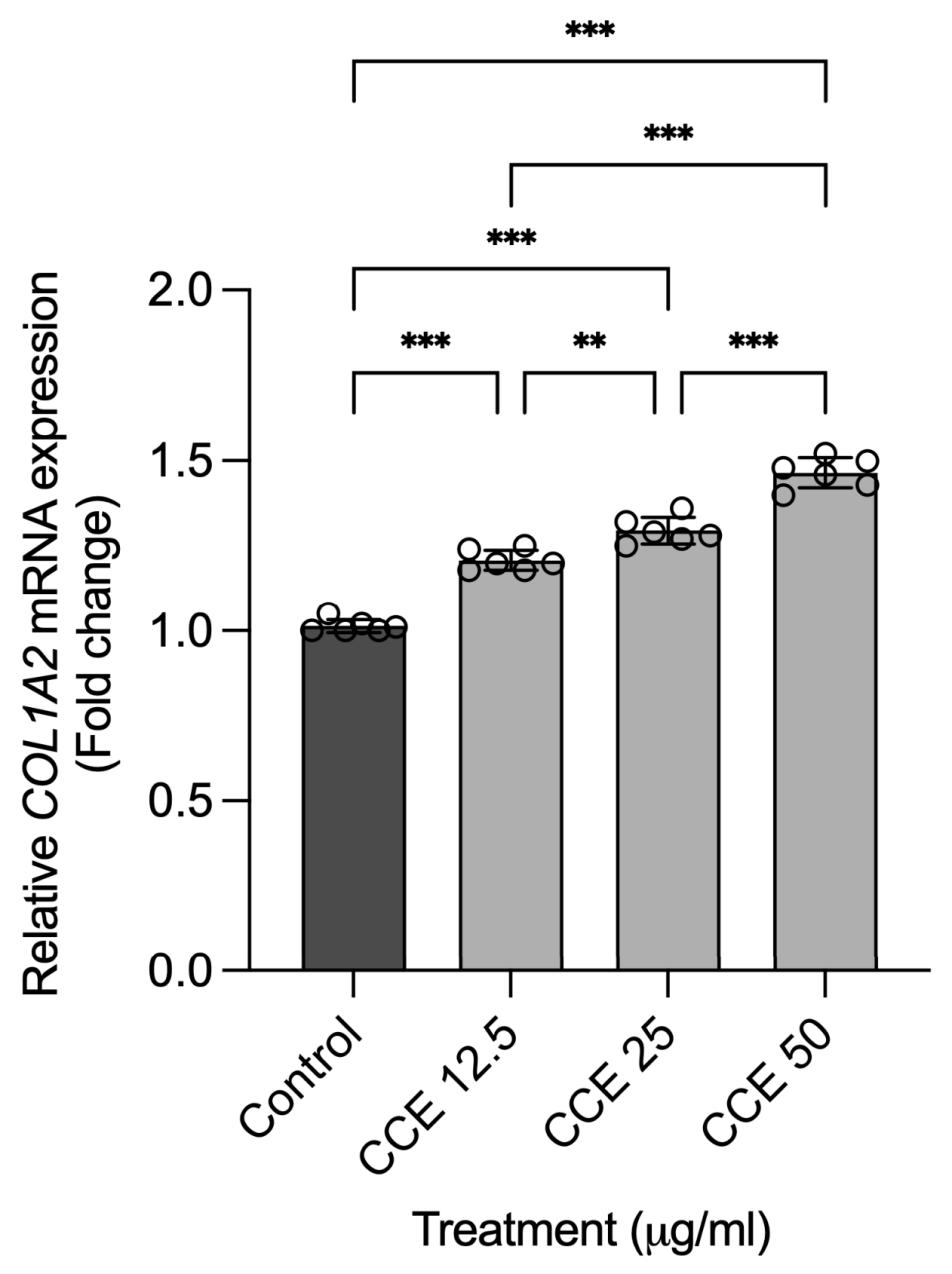
Relative mRNA expression of *COL1A2* in UV-irradiated human dermal fibroblasts treated with the selected *Chara corallina* extract. Relative expression was determined by one-step RT-qPCR and normalized to *GAPDH*. Data are presented as mean ± SD from three independent experiments (*n* = 3). White circles represent individual data points. Technical replicate measurements within each independent experiment were averaged before statistical analysis. Statistical significance between the indicated groups was analyzed by one-way ANOVA followed by Tukey’s multiple comparison test. *p* < 0.01 (**), and *p* < 0.001 (***).

The effect of the selected extract on collagen-related gene expression was evaluated by measuring *COL1A2* expression in UV-irradiated human dermal fibroblasts. As shown in Figure 6, treatment with the selected CCE significantly increased *COL1A2* expression in a concentration-dependent manner compared with the UV-irradiated control (*p* < 0.05).

Relative *COL1A2* expression increased from 1.01 ± 0.01 in the UV-treated control to 1.21 ± 0.02, 1.30 ± 0.03, and 1.47 ± 0.04 following treatment with 12.5, 25, and 50 μg mL^−1^, respectively. The highest expression level was observed at 50 μg mL^−1^.

These findings demonstrate that the selected CCE significantly enhanced *COL1A2* expression under UV-induced oxidative stress conditions.

#### 3.5.2. Inhibition of Extracellular Matrix-Degrading Enzymes

**Figure 7:**
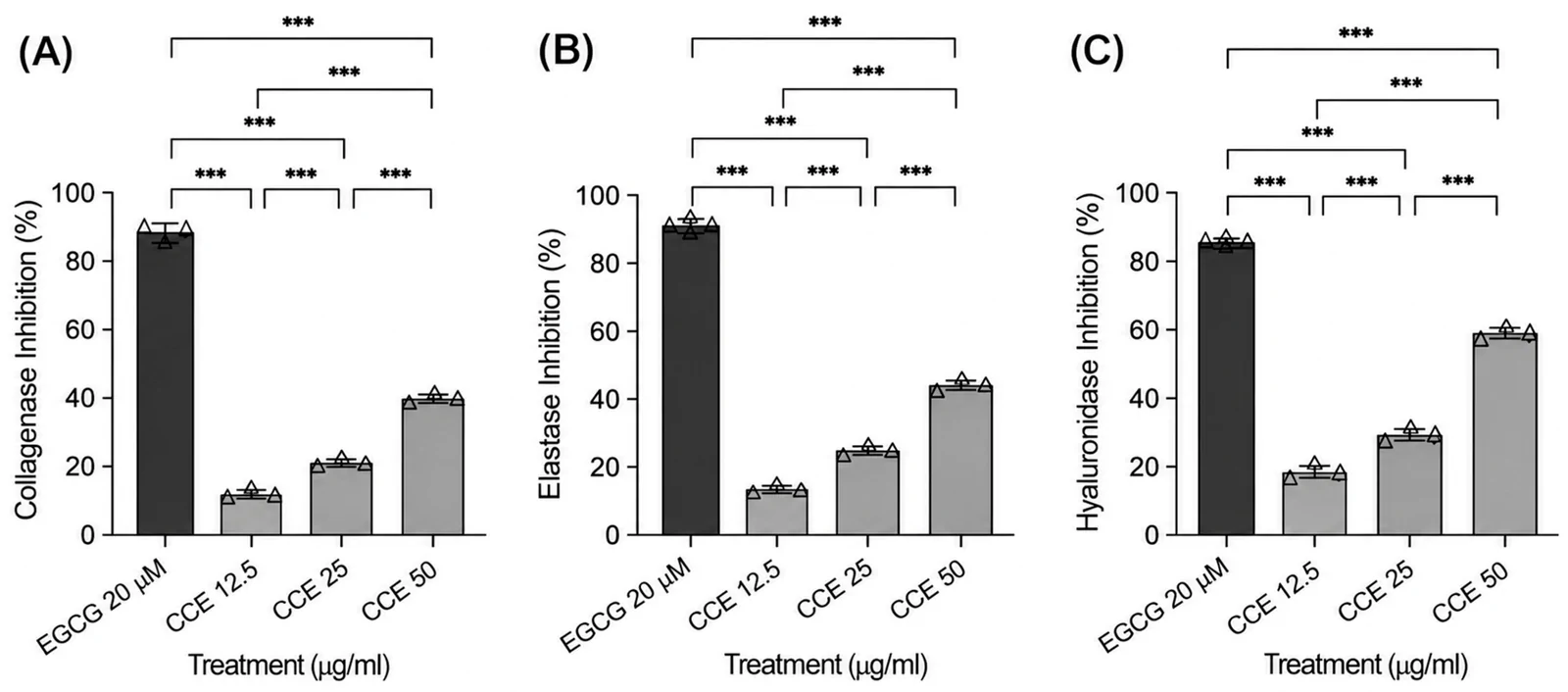
Inhibitory effects of CCE on extracellular matrix-degrading enzymes: (**A**) collagenase, (**B**) elastase, and (**C**) hyaluronidase. EGCG (20 μM) was used as the positive inhibitory control. Data are presented as mean ± SD from three independent experiments (*n* = 3). White triangles represent individual data points. Technical replicate measurements within each independent experiment were averaged before statistical analysis. Statistical significance between the indicated groups was analyzed by one-way ANOVA followed by Tukey’s multiple comparison test. *p* < 0.001 (***).

CCE exhibited inhibition of collagenase, elastase, and hyaluronidase across the evaluated concentration range of 12.5–50 μg mL^−1^. EGCG (20 μM) was included as the positive inhibitory control for all three enzyme assays (Figure 7). The extract inhibited all three enzymes in a concentration-dependent manner, with the greatest inhibitory activity observed at the highest tested concentration.

For collagenase (Figure 7A), inhibitory activity increased from 10.84 ± 1.50% at 12.5 μg mL^−1^ to 20.12 ± 1.83% and 38.69 ± 1.76% at 25 and 50 μg mL^−1^, respectively. Similarly, elastase inhibition (Figure 7B) increased from 13.23 ± 1.34% to 24.50 ± 1.67% and 44.17 ± 2.08%, whereas hyaluronidase inhibition (Figure 7C) increased from 17.53 ± 2.01% to 28.48 ± 2.21% and 57.24 ± 2.15% across the same concentration range. All treatment groups exhibited significantly greater inhibitory activity than the untreated control (*p* < 0.05).

The positive control, EGCG (20 μM), exhibited higher inhibitory activity against collagenase (87.87 ± 2.44%), elastase (90.38 ± 2.36%), and hyaluronidase (86.41 ± 2.00%) than the selected extract.

Collectively, these findings demonstrate that the selected CCE enhanced *COL1A2* expression and inhibited the activities of collagenase, elastase, and hyaluronidase in a concentration-dependent manner.

## 4. Discussion

### 4.1. Astaxanthin-Equivalent Recovery Under the Selected Ultrasound-Assisted Extraction Conditions

The present study evaluated astaxanthin-equivalent recovery from the freshwater macroalga *Chara corallina* under defined ultrasound-assisted extraction (UAE) conditions. Among the solvent systems examined, 48% ethanol in ethyl acetate provided the highest astaxanthin-equivalent recovery, yielding approximately 2.98-fold and 1.33-fold higher values than ethanol and ethyl acetate alone, respectively. HPLC–PDA analysis of the selected extract further revealed a chromatographic component with retention-time and UV–visible spectral characteristics corresponding to those of the authentic astaxanthin reference standard.

The extraction pattern observed in the present study is consistent with the physicochemical principles underlying UAE. During ultrasonication, acoustic cavitation generates microscopic bubbles that collapse rapidly, producing localized shear forces and microjets that can disrupt cellular structures, enhance solvent penetration, and facilitate mass transfer, thereby promoting the recovery of intracellular carotenoids [33,34]. The relatively mild extraction conditions employed in the present study (30 °C for 15 min) are also consistent with conditions generally considered favorable for limiting excessive thermal exposure of carotenoids during extraction [35,36]. However, the present analytical approach does not establish preservation of astaxanthin chemical integrity. Although HPLC–PDA revealed a chromatographic component showing retention-time and UV–visible spectral correspondence with the authentic astaxanthin reference standard, the additional chromatographic peaks observed in CCE were not structurally characterized. These unresolved components may represent other carotenoids, astaxanthin derivatives, or other extract constituents, and the possibility that some represent degradation or oxidation products cannot be excluded. Consequently, the present data do not demonstrate that UAE preserved the chemical integrity, stability, or antioxidant functionality of astaxanthin during extraction. Orthogonal structural characterization, particularly LC–MS/MS, would be required to identify these components, distinguish related carotenoids and astaxanthin derivatives, and investigate potential degradation products.

In addition to ultrasonic processing, solvent polarity likely contributed to the differences in recovery among the solvent systems examined. Astaxanthin contains a hydrophobic conjugated polyene chain together with polar hydroxyl and keto groups, and its extraction is therefore strongly influenced by solvent polarity and interactions with the biomass matrix [37,38]. The selected ethanol–ethyl acetate system may have provided a favorable polarity balance for carotenoid recovery from *C. corallina*. Similar effects of solvent composition on carotenoid recovery have been reported for various algal matrices [6,39,40,41]. Ultrasound-assisted extraction has likewise been investigated for the recovery of carotenoids and other antioxidant compounds from microalgae and marine macroalgae, where improved mass transfer and disruption of cellular structures can facilitate extraction [34,36,42]. Nevertheless, most astaxanthin-focused UAE studies have involved microalgae, particularly *Haematococcus pluvialis*, whereas comparable information for freshwater macroalgae remains limited [38,43,44].

For quantitative context, the highest astaxanthin-equivalent yield obtained from *C. corallina* in the present study was 0.2598 ± 0.0086% of dry biomass. This value is substantially lower than the astaxanthin contents reported for *H. pluvialis*, the principal commercial biological source of natural astaxanthin. Previous studies have reported astaxanthin concentrations of approximately 2.7–4.0% of dry biomass in *H. pluvialis*, with values approaching 5% under favorable cultivation and stress conditions [4,45]. Thus, the value observed for *C. corallina* in the present study is approximately one order of magnitude lower than the levels commonly reported for astaxanthin-accumulating *H. pluvialis*. Direct quantitative comparison should nevertheless be interpreted cautiously because reported astaxanthin contents and recoveries are strongly influenced by species and strain, cultivation and stress conditions, physiological stage, biomass pretreatment, extraction procedure, solvent system, and analytical methodology. Accordingly, the present findings do not indicate that *C. corallina* is quantitatively competitive with *H. pluvialis* as a source of natural astaxanthin.

The present findings nevertheless extend the limited quantitative information available for *C. corallina* by demonstrating measurable astaxanthin-equivalent recovery under the UAE conditions evaluated in this study. The chemical findings should, however, be interpreted within the analytical scope of the study. HPLC–PDA provided quantitative information for a chromatographic component corresponding to the authentic astaxanthin reference standard, but orthogonal structural confirmation was not performed, and the complete carotenoid and phytochemical composition of CCE was not established. The present data are therefore insufficient to position *C. corallina* as a chemically defined, quantitatively competitive, or commercially established source of natural astaxanthin. Rather, the contribution of the present study lies in the integration of UAE-based recovery, HPLC–PDA-based assessment of an astaxanthin-corresponding component, and biological evaluation of the resulting chemically complex extract from an underexplored freshwater macroalgal biomass. The findings should therefore be interpreted as an initial evaluation of a carotenoid-containing bioactive extract from *C. corallina*, rather than as evidence establishing this species as an alternative to currently marketed natural astaxanthin sources.

This distinction is also important for interpretation of the biological findings. The selected CCE represents a chemically complex algal extract rather than purified astaxanthin, and purified astaxanthin was not included as a comparator in the biological assays. Therefore, the DPPH radical-scavenging activity, modulation of antioxidant- and inflammation-associated gene expression, increased *COL1A2* mRNA expression, and inhibition of extracellular matrix-degrading enzymes observed in the present study should be interpreted as properties of CCE as a whole and cannot be attributed exclusively to astaxanthin. Other carotenoids and unidentified constituents may have contributed independently or through additive or synergistic interactions. Moreover, because biological activities were not directly compared among extracts obtained using different solvent systems or with a conventional non-UAE extraction method, the present data do not establish that UAE itself preserved or enhanced biological functionality. Accordingly, previously reported molecular and biological activities of purified astaxanthin are discussed below only as relevant biological context rather than as direct mechanistic evidence for the activity of CCE.

### 4.2. Antioxidant-Related Activity of the Selected CCE

The selected CCE exhibited antioxidant-related activity at both chemical and cellular endpoints. In the cell-free assay, CCE demonstrated DPPH radical-scavenging activity, indicating its ability to scavenge DPPH radicals under the experimental conditions employed. However, because DPPH was the only cell-free antioxidant assay performed, these findings should be interpreted specifically as evidence of DPPH radical-scavenging activity rather than as demonstrating broad antioxidant capacity. At the cellular level, CCE significantly increased the mRNA expression of the antioxidant-related genes *SOD1*, *CAT*, and *GPX1*, providing complementary evidence of a transcriptional response associated with antioxidant defense. Nevertheless, these changes in gene expression do not necessarily indicate corresponding increases in antioxidant enzyme abundance or activity, and intracellular reactive oxygen species were not directly measured. Therefore, the present findings support the DPPH radical-scavenging activity of CCE together with its ability to modulate antioxidant-related gene expression, while further studies incorporating additional chemical antioxidant assays and direct measurements of cellular oxidative stress and antioxidant enzyme activity are required to establish its broader antioxidant effects.

The DPPH radical-scavenging activity observed in the present study is consistent with the well-established antioxidant properties of astaxanthin. Owing to its extended conjugated polyene chain and terminal hydroxyl and keto groups, astaxanthin can quench singlet oxygen and scavenge free radicals [37,38]. These structural characteristics contribute to its antioxidant properties relative to many other naturally occurring carotenoids [38,46]. However, CCE represents a complex algal extract rather than purified astaxanthin, and the observed DPPH radical-scavenging activity therefore cannot be attributed exclusively to astaxanthin. Other carotenoids or unidentified constituents of the extract may also have contributed to the observed activity. Furthermore, because biological activity was not directly compared among extracts obtained using different solvent systems or with a conventional non-UAE extraction method, the present data do not establish that UAE itself preserved or enhanced biological functionality. Accordingly, the observed antioxidant-related responses should be interpreted as properties of the selected CCE rather than as evidence of a UAE-mediated preservation effect. Direct comparative studies using appropriately matched extraction methods would be required to determine whether the extraction approach influences the biological activity of the resulting extracts.

Beyond its direct radical-scavenging properties, astaxanthin has been reported to enhance endogenous antioxidant defenses by modulating cellular redox homeostasis [38,46]. Previous studies have suggested that these effects may involve activation of the nuclear factor erythroid 2-related factor 2 (Nrf2)–antioxidant response element (ARE) signaling pathway, which regulates the transcription of antioxidant genes encoding enzymes such as superoxide dismutase, catalase, and glutathione peroxidase [46,47]. In the present study, CCE increased the mRNA expression of *SOD1*, *CAT*, and *GPX1*. Similar transcriptional responses have been reported in studies of astaxanthin and have been associated with Nrf2-regulated antioxidant pathways [46,47]. These previous findings provide a possible mechanistic context for the responses observed with CCE; however, they do not demonstrate that astaxanthin was solely responsible for the present effects or that Nrf2 signaling was activated by CCE. However, because Nrf2 activation was not directly evaluated at the protein level or through nuclear translocation assays, these findings should not be interpreted as direct evidence of Nrf2 activation. Future studies incorporating protein expression analysis, assessment of Nrf2 nuclear translocation, or functional pathway inhibition will be necessary to verify the upstream signaling mechanisms responsible for the observed transcriptional responses.

The coordinated upregulation of *SOD1*, *CAT*, and *GPX1* also has important biological implications. These enzymes function cooperatively to detoxify reactive oxygen species and maintain intracellular redox homeostasis, thereby limiting oxidative damage to cellular macromolecules [48]. Similar coordinated transcriptional responses have previously been reported following astaxanthin treatment in both microalgal extracts and purified astaxanthin preparations [46,47], supporting the biological relevance of the antioxidant response observed in the present study.

Collectively, the present findings demonstrate DPPH radical-scavenging activity together with modulation of antioxidant-related gene expression by the selected CCE. These complementary chemical and transcriptional observations support the antioxidant-related potential of the extract but do not establish the underlying cellular signaling mechanisms or functional enhancement of endogenous antioxidant enzyme activity. The findings are consistent with previous evidence describing antioxidant-related and skin-protective effects of astaxanthin and other carotenoids [49,50]. However, given the complex composition of CCE, the observed responses cannot be attributed exclusively to astaxanthin, and further chemical characterization and mechanistic studies are required to identify the constituents responsible for these effects.

### 4.3. Anti-Inflammatory Activity Through Modulation of Inflammation-Associated Genes

The present study demonstrated that the selected CCE significantly downregulated the expression of the inflammation-associated genes *Nos2*, *Ptgs2*, and *Il1b* in LPS-stimulated RAW264.7 macrophages without affecting cell viability. The coordinated suppression of these genes suggests attenuation of macrophage inflammatory responses and indicates that the extract modulates multiple inflammatory mediators rather than targeting a single signaling component. Together with the cytocompatibility observed in RAW264.7 cells, these findings indicate that the anti-inflammatory effects were attributable to biological activity of the extract rather than nonspecific cytotoxicity.

The anti-inflammatory effects observed in the present study are consistent with previous reports describing the immunomodulatory properties of astaxanthin and astaxanthin-rich extracts [38,46]. Astaxanthin has been proposed to attenuate inflammatory responses by reducing oxidative stress and modulating redox-sensitive signaling pathways, particularly those involving nuclear factor-κB (NF-κB), which regulates the transcription of numerous pro-inflammatory genes [47,51]. Because *Nos2*, *Ptgs2*, and *Il1b* are well-recognized downstream targets of NF-κB signaling, the coordinated downregulation of these genes observed in the present study is compatible with previous studies describing NF-κB-associated anti-inflammatory effects of astaxanthin. However, because NF-κB activation was not directly evaluated in the present study, these findings should not be interpreted as direct evidence of NF-κB inhibition but rather suggest the possible involvement of this signaling pathway. Future studies incorporating analysis of NF-κB nuclear translocation, phosphorylation status, or functional inhibition assays will be required to verify the upstream signaling mechanisms responsible for the observed transcriptional responses.

The coordinated downregulation of *Nos2*, *Ptgs2*, and *Il1b* is biologically relevant because these genes represent key mediators of macrophage inflammatory activation. *Nos2* encodes inducible nitric oxide synthase, whereas *Ptgs2* encodes cyclooxygenase-2, both of which are rapidly induced during inflammatory stimulation and contribute to the production of inflammatory mediators [51,52]. Likewise, *Il1b* encodes interleukin-1β, an important cytokine that amplifies inflammatory responses and promotes recruitment of immune cells [53]. Simultaneous suppression of these three genes therefore indicates broad attenuation of inflammatory signaling rather than inhibition of a single inflammatory pathway.

The present findings are in agreement with previous studies demonstrating that astaxanthin suppresses inflammation-associated gene expression in activated macrophages and other experimental models of inflammation [38,46,47]. Nevertheless, most previous investigations have focused on purified astaxanthin or extracts derived from the microalga *Haematococcus pluvialis*. In contrast, comparatively little information is available regarding the anti-inflammatory activity of astaxanthin-rich extracts obtained from freshwater macroalgae. The present study therefore extends current knowledge by demonstrating that an astaxanthin-rich extract prepared from *Chara corallina* exhibits anti-inflammatory activity, highlighting the potential of this underexplored freshwater macroalga as an alternative source of bioactive carotenoids.

Collectively, CCE reduced the expression of the inflammation-associated genes *Nos2*, *Ptgs2*, and *Il1b* relative to the LPS-stimulated vehicle control. Although similar effects have been reported for purified astaxanthin, the present findings should be interpreted as CCE-associated transcriptional responses rather than as direct evidence of an astaxanthin-specific mechanism. Moreover, because an unstimulated normal control and direct measurements of inflammatory proteins or signaling pathways were not included, the magnitude of the LPS-induced response and the underlying molecular mechanisms could not be established.

### 4.4. Extracellular Matrix-Related Effects Relevant to Photoaging

The present study demonstrated that the selected CCE increased *COL1A2* mRNA expression and inhibited collagenase, elastase, and hyaluronidase within the experimentally evaluated concentration range. Previous studies have reported effects of purified astaxanthin on pathways associated with collagen homeostasis and photoaging [53,54]. However, because purified astaxanthin was not tested in parallel with CCE, these previous findings provide contextual support rather than direct evidence that astaxanthin was responsible for the *COL1A2* response or enzyme-inhibitory activities observed in the present study. Moreover, increased *COL1A2* mRNA expression does not necessarily indicate increased collagen protein synthesis or extracellular matrix deposition.

Type I collagen is the predominant structural protein of the dermal extracellular matrix and plays an essential role in maintaining skin strength, elasticity, and mechanical integrity [55]. During photoaging, ultraviolet radiation suppresses collagen gene expression while simultaneously accelerating extracellular matrix degradation, ultimately leading to dermal thinning and wrinkle formation [53,54]. The significant upregulation of *COL1A2* observed in the present study therefore indicates enhanced collagen-associated transcriptional activity following treatment with the selected extract. Nevertheless, because collagen protein expression and extracellular matrix deposition were not directly determined, these findings should be interpreted as evidence of increased collagen biosynthetic potential at the transcriptional level rather than direct confirmation of collagen synthesis. Future studies evaluating collagen protein expression, hydroxyproline content, or extracellular matrix deposition will be required to verify these downstream biological effects.

Besides modulating collagen-associated gene expression, the selected extract significantly inhibited collagenase, elastase, and hyaluronidase activities. These enzymes are major contributors to extracellular matrix remodeling during skin aging, and their excessive activation accelerates degradation of collagen, elastin, and hyaluronic acid, thereby compromising skin structure, elasticity, and hydration [56,57]. The simultaneous inhibition of all three enzymes observed in the present study therefore suggests broad protection of multiple extracellular matrix components rather than selective preservation of collagen alone. Moreover, the concentration-dependent inhibition of these enzymes is consistent with the multifunctional biological properties expected of natural anti-photoaging compounds.

The present findings are consistent with previous experimental studies and recent reviews demonstrating that astaxanthin improves skin health through multiple complementary mechanisms, including modulation of collagen metabolism, attenuation of oxidative stress, and protection against extracellular matrix degradation [38,46,57]. Recent comprehensive reviews further emphasize that carotenoids, particularly astaxanthin, preserve skin integrity through coordinated regulation of antioxidant defenses, inflammatory responses, and extracellular matrix homeostasis, supporting the biological relevance of the present findings [50]. However, most previous investigations have evaluated purified astaxanthin or extracts derived from the microalga *Haematococcus pluvialis*. In contrast, evidence regarding astaxanthin-rich extracts obtained from freshwater macroalgae remains very limited. The present study therefore extends current knowledge by demonstrating that an astaxanthin-rich extract prepared from *Chara corallina* exhibits anti-photoaging activity, highlighting this underexplored freshwater macroalga as a promising alternative source of naturally derived bioactive carotenoids.

Collectively, the increased *COL1A2* mRNA expression and inhibition of extracellular matrix-degrading enzymes support the potential relevance of CCE to extracellular matrix-related processes associated with skin photoaging. However, because no direct comparator was included for the overall anti-photoaging response, the present findings do not establish comparable or superior anti-photoaging activity relative to purified astaxanthin or other established bioactive compounds. Moreover, increased *COL1A2* expression should not be interpreted as direct evidence of increased collagen protein synthesis or extracellular matrix deposition. Accordingly, the present results support the anti-photoaging potential of CCE rather than comparative anti-photoaging efficacy.

### 4.5. Proposed Mechanism and Biological Significance

The collective findings of the present study support a biologically integrated framework linking antioxidant, anti-inflammatory, and anti-photoaging activities of the selected CCE obtained from *Chara corallina*. Rather than representing independent biological effects, the observed responses appear to reflect coordinated regulation of cellular processes associated with skin protection. The selected extract exhibited DPPH radical-scavenging activity together with enhanced expression of endogenous antioxidant genes, suppression of inflammation-associated genes, increased *COL1A2* expression, and inhibition of extracellular matrix-degrading enzymes. Collectively, these complementary responses suggest preservation of skin cellular homeostasis following treatment with the selected extract.

**Figure 8:**
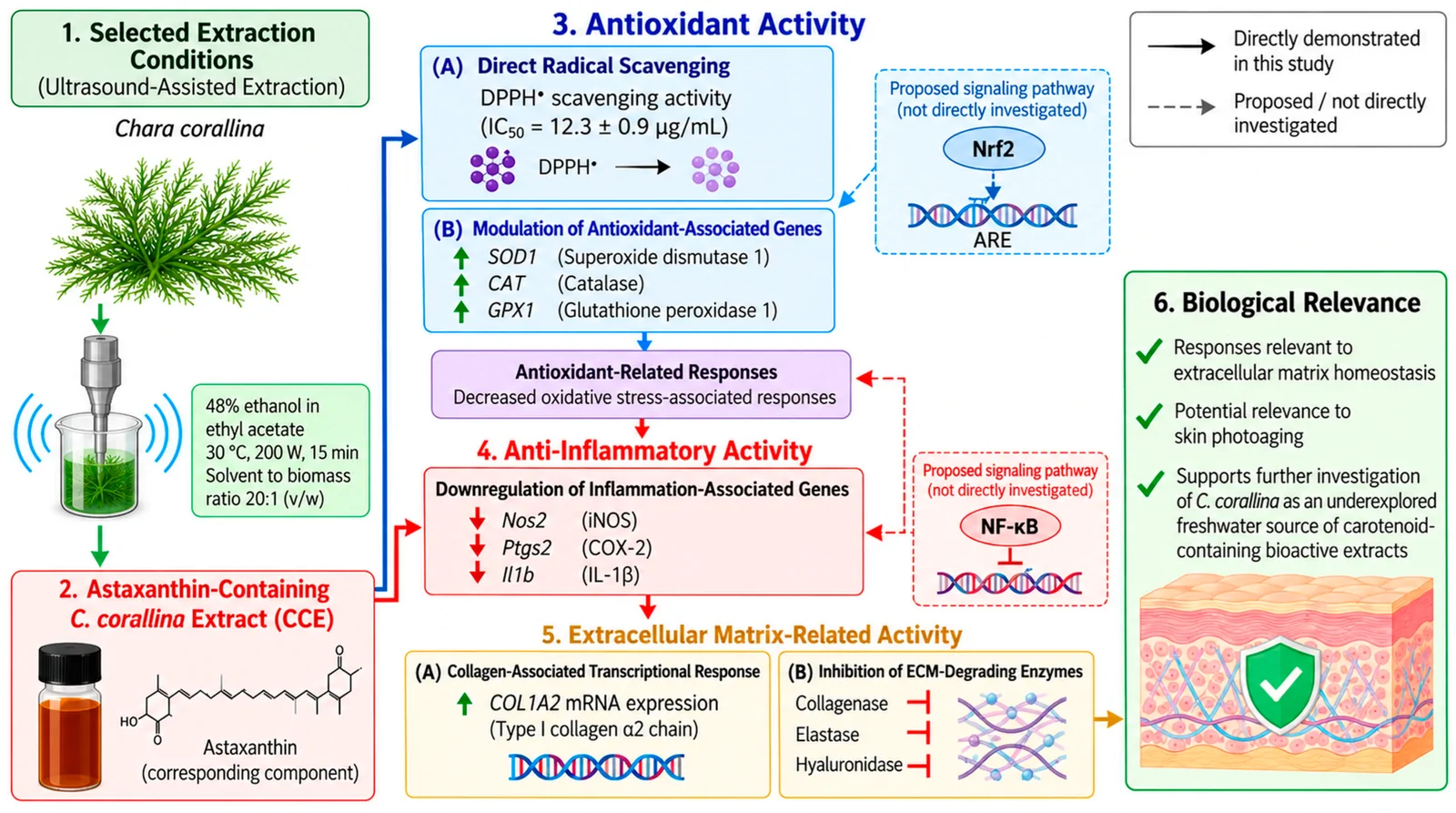
Proposed biological framework summarizing the experimentally observed responses associated with the selected *Chara corallina* extract (CCE). Under the evaluated UAE conditions, the selected solvent system provided the highest astaxanthin-equivalent recovery among the solvent systems examined. CCE exhibited DPPH radical-scavenging activity, modulated antioxidant- and inflammation-associated gene expression, increased *COL1A2* mRNA expression, and inhibited collagenase, elastase, and hyaluronidase. Because CCE is a chemically complex extract, these biological responses cannot be attributed exclusively to astaxanthin. Dashed arrows indicate proposed biological relationships or signaling pathways that were not directly investigated in the present study.

A plausible biological framework underlying these observations is illustrated in Figure 8. The selected extract demonstrated potent DPPH radical scavenging activity and significantly upregulated *SOD1*, *CAT*, and *GPX1*, indicating enhancement of endogenous antioxidant defenses. Previous studies have suggested that astaxanthin may regulate these responses through redox-sensitive signaling pathways, particularly the Nrf2–ARE pathway, which controls the transcription of numerous cytoprotective genes [38,46,47]. In parallel, the coordinated downregulation of *Nos2*, *Ptgs2*, and *Il1b* observed in LPS-stimulated macrophages is compatible with attenuation of inflammatory responses and may involve modulation of NF-κB-associated signaling [47,51,52]. However, because neither Nrf2 nor NF-κB signaling was directly evaluated in the present study, the involvement of these pathways should be regarded as a biologically plausible mechanism supported by the previous literature rather than definitive evidence [49].

The coordinated enhancement of antioxidant defenses together with attenuation of inflammatory gene expression may contribute to preservation of dermal extracellular matrix homeostasis. Consistent with this concept, the selected extract significantly increased *COL1A2* expression while simultaneously inhibiting collagenase, elastase, and hyaluronidase activities. These complementary responses suggest maintenance of extracellular matrix integrity through enhancement of collagen-associated transcription together with reduced enzymatic degradation of structural matrix components [53,54,55,56,57,58,59,60]. Nevertheless, because collagen protein expression, extracellular matrix deposition, and upstream signaling pathways were not directly evaluated, these findings should be interpreted as mechanistically supportive rather than conclusive.

Collectively, the present findings demonstrate that the selected CCE exhibits biological responses relevant to oxidative stress, inflammation, and extracellular matrix-related processes, including DPPH radical-scavenging activity, modulation of antioxidant- and inflammation-associated gene expression, increased *COL1A2* mRNA expression, and inhibition of collagenase, elastase, and hyaluronidase. These processes are biologically relevant to skin photoaging, in which oxidative stress, persistent inflammatory signaling, and extracellular matrix degradation are closely interconnected [49,50]. Previous studies and reviews have similarly described purified astaxanthin as a carotenoid capable of modulating antioxidant, inflammatory, and extracellular matrix-related pathways [49,50]. However, these reports provide biological context rather than direct mechanistic evidence for the activity of CCE because the present extract is chemically complex and purified astaxanthin was not evaluated as a comparator. Moreover, the present transcriptional and enzyme-inhibition findings do not directly demonstrate extracellular matrix preservation or protection against photoaging. Accordingly, the current results support further investigation of CCE as a carotenoid-containing bioactive extract with biological activities relevant to skin photoaging. However, comprehensive chemical characterization, mechanistic validation, evaluation in more physiologically relevant skin models, and subsequent formulation, stability, safety, and skin-penetration studies will be required before its potential cosmeceutical applicability can be established.

### 4.6. Study Limitations and Future Perspectives

Several limitations should be considered when interpreting the present findings. First, all *Chara corallina* biomass used in this study was obtained from a single freshwater pond during a single sampling period in March 2025. Because carotenoid composition and accumulation may vary with season, geographical location, environmental conditions, and physiological state, the astaxanthin-equivalent recovery and extract composition reported here should be regarded as representative of the specific biomass batch examined rather than as species-wide values for *C. corallina*. Moreover, the three extraction replicates were independently processed aliquots derived from the same collected biomass batch and therefore represent independent extraction replicates rather than independent field-level biological replicates. Consequently, the reported variability primarily reflects extraction-level variation and does not capture spatial, temporal, or population-level biological variability. Future studies incorporating independently collected biomass from multiple locations and seasons will be required to establish the natural variability and reproducibility of carotenoid recovery from this species.

Second, the study did not include a conventional non-UAE extraction control or a direct experimental comparison with established commercial astaxanthin sources such as *Haematococcus pluvialis*. The present results therefore allow comparison among the solvent systems evaluated under the applied UAE conditions but do not establish the superiority of UAE over conventional extraction or the quantitative competitiveness of *C. corallina* with established astaxanthin sources. In addition, the chemical characterization of CCE was limited to HPLC–PDA comparison with an authentic astaxanthin reference standard. Although a chromatographic component showed retention-time and UV–visible spectral correspondence with the authentic astaxanthin reference standard, orthogonal structural confirmation by LC–MS/MS or another mass-spectrometric technique was not performed. Therefore, definitive structural identification, differentiation of free and esterified forms or *E*/*Z* isomers, and comprehensive characterization of other carotenoids were beyond the analytical scope of the present study. Moreover, the additional chromatographic peaks observed in CCE cannot presently be assigned to other carotenoids, astaxanthin derivatives, or potential degradation or oxidation products. Consequently, preservation of astaxanthin chemical integrity or stability during UAE cannot be concluded from the present data. Future studies employing comprehensive LC–MS/MS-based profiling together with chromatographic methods optimized for carotenoid, isomer, and ester characterization will be required to resolve these components and more comprehensively define the chemical composition of CCE. Accordingly, the present study does not establish *C. corallina* as a chemically defined or commercially competitive source of natural astaxanthin.

Third, CCE is a chemically complex algal extract rather than purified astaxanthin, and purified astaxanthin was not included as a comparator in the biological assays. Consequently, the observed DPPH radical-scavenging, gene-expression, and extracellular matrix-related enzyme-inhibitory effects cannot be attributed exclusively to astaxanthin. Other carotenoids and unidentified constituents may have contributed independently or through additive or synergistic interactions. Furthermore, antioxidant activity was evaluated using DPPH as the only cell-free chemical assay, while cellular antioxidant-related responses were assessed at the transcriptional level without direct measurements of intracellular reactive oxygen species, antioxidant protein abundance, or enzyme activity. Similarly, inflammation-related responses were evaluated primarily through changes in *Nos2*, *Ptgs2*, and *Il1b* mRNA expression without corresponding protein-level measurements of inflammatory mediators or direct assessment of upstream signaling pathways. Thus, proposed mechanisms involving pathways such as Nrf2–ARE or NF-κB should be regarded as biologically plausible interpretations based on the previous literature rather than as mechanisms directly demonstrated in the present study.

Fourth, additional limitations apply to the cellular and extracellular matrix-related experiments. The relatively broad HDF passage range (passages 5–20) may have introduced passage-dependent variation in fibroblast phenotype and transcriptional responses. In addition, the UV-irradiation and LPS-stimulation experiments did not include corresponding nonirradiated or unstimulated normal controls. Therefore, the magnitude of UV- or LPS-induced transcriptional changes relative to a baseline could not be determined, and the observed gene-expression responses should be interpreted as CCE-associated changes relative to the corresponding UV-irradiated or LPS-stimulated vehicle controls rather than as restoration toward normal expression levels. Increased *COL1A2* mRNA expression likewise does not directly demonstrate increased collagen protein synthesis or extracellular matrix deposition. Moreover, although CCE inhibited collagenase, elastase, and hyaluronidase within the experimentally evaluated concentration range, these cell-free enzyme assays do not directly establish preservation of extracellular matrix integrity in a biological skin model.

Finally, the biological evaluation was conducted using cell-free assays and conventional two-dimensional cell culture models, which do not fully reproduce the complexity of human skin physiology, including tissue architecture, multicellular interactions, compound penetration, metabolism, and bioavailability. Environmental sustainability, biomass supply, commercial feasibility, formulation stability, skin penetration, and long-term safety were also not experimentally evaluated in the present study. Accordingly, sustainability and practical cosmeceutical applicability cannot yet be established from the present findings. Future investigations should prioritize independent multi-site and seasonal biomass sampling, comprehensive chemical profiling and identification of active constituents, direct comparison with purified astaxanthin and appropriately matched extraction controls, functional and protein-level mechanistic validation, and evaluation in more physiologically relevant models such as three-dimensional skin equivalents or ex vivo skin. Subsequent formulation, stability, safety, environmental, and techno-economic assessments will be necessary to determine the feasibility of practical cosmeceutical development.

## 5. Conclusions

This study evaluated UAE-based recovery and biological properties of a carotenoid-containing extract from the freshwater macroalga *Chara corallina*. Among the solvent systems examined, 48% ethanol in ethyl acetate provided the highest astaxanthin-equivalent recovery. HPLC–PDA analysis revealed a chromatographic component with retention-time and UV–visible spectral characteristics corresponding to those of an authentic astaxanthin reference standard, although orthogonal structural confirmation and comprehensive carotenoid profiling were not performed.

The selected CCE exhibited DPPH radical-scavenging activity, modulated antioxidant- and inflammation-associated gene expression, increased *COL1A2* mRNA expression, and inhibited extracellular matrix-degrading enzymes within the evaluated concentration range. Because CCE is a chemically complex extract and purified astaxanthin was not included as a biological comparator, these responses cannot be attributed exclusively to astaxanthin.

Overall, the findings provide initial evidence supporting further investigation of *C. corallina* as an underexplored freshwater biomass for carotenoid-containing bioactive extracts. However, the present data do not establish *C. corallina* as a chemically defined, quantitatively competitive, or commercially viable source of natural astaxanthin. Comprehensive structural characterization, independent biomass validation, and appropriately controlled biological studies will be required before such conclusions can be established.

## Figures and Tables

**Table 1:** Astaxanthin-equivalent recovery from *Chara corallina* using different solvent systems under ultrasound-assisted extraction conditions. Each independent extraction replicate was performed using 52.5 g of dried biomass at a solid-to-solvent ratio of 1:20 (*w*/*v*), with two sequential extraction cycles performed under the same conditions.

Extraction Solvent	Astaxanthin-Equivalent Recovery (mg)	Astaxanthin-Equivalent Yield (% *w*/*w*)
Ethanol	45.8 ± 2.1	0.0873 ± 0.0042
Ethyl acetate	102.5 ± 3.7	0.1952 ± 0.0071
48% ethanol in ethyl acetate	136.4 ± 4.5	0.2598 ± 0.0086

Each independent extraction replicate consisted of two sequential extraction cycles using 52.5 g of dried *C. corallina* biomass at a solid-to-solvent ratio of 1:20 (*w*/*v*). Following the first extraction, the residual biomass was re-extracted with fresh solvent of the same composition under the same extraction conditions, and the resulting supernatants were combined. Astaxanthin-equivalent recovery was quantified by HPLC–PDA using an external calibration curve prepared from an authentic astaxanthin reference standard. No specific free, esterified, or *E*/*Z* isomeric form was assigned. Data are expressed as mean ± SD of three independent extraction replicates (*n* = 3), each prepared separately from aliquots of the same collected biomass batch.

**Table 2:** DPPH radical-scavenging activity of the selected *Chara corallina* extract (CCE) and Trolox as the reference antioxidant.

Sample	IC_50_ (μg/mL)
Trolox	7.3 ± 0.8
Selected *Chara corallina* extract	12.3 ± 0.9

## Data Availability

The data presented in this study are available on request from the corresponding author due to privacy and personal constraints of the research team.

## Supplementary Materials

The following supporting information can be downloaded at https://www.mdpi.com/article/10.3390/antiox15091159/s1, Table S1: Primer sequences used for one-step reverse transcription quantitative PCR (RT-qPCR). Table S2: RT-qPCR assay information. Figure S1: Calibration curve of the authentic astaxanthin standard analyzed by HPLC–PDA at 476 nm.

## Abbreviations

The following abbreviations are used in this manuscript:

UAE	Ultrasound-Assisted Extraction
HPLC	High-Performance Liquid Chromatography
DPPH	2,2-diphenyl-1-picrylhydrazyl
ECM	Extracellular Matrix
PTFE	Polytetrafluoroethylene
PDA	Photodiode Array
% *w*/*w*	Percentage Weight by Weight
HDFs	Human Dermal Fibroblasts
ATCC	American Type Culture Collection
RAW264.7	RAW 264.7 Cell Line
LPS	Lipopolysaccharide
DMEM	Dulbecco’s Modified Eagle Medium
FBS	Fetal Bovine Serum
RPMI-1640	Roswell Park Memorial Institute 1640 Medium
DMSO	Dimethyl Sulfoxide
MTT	3-(4,5-dimethylthiazol-2-yl)-2,5-diphenyltetrazolium bromide
UVA	Ultraviolet A
UVB	Ultraviolet B
MIQE	Minimum Information for Publication of Quantitative Real-Time PCR Experiments
EGCG	Epigallocatechin Gallate
Trolox	6-hydroxy-2,5,7,8-tetramethylchroman-2-carboxylic acid
CCE	*Chara corallina* Extract
ROS	Reactive Oxygen Species
Nrf2	Nuclear factor erythroid 2-related factor 2
ARE	Antioxidant Response Element
NF-κB	Nuclear Factor Kappa B
LC-MS/MS	Liquid Chromatography–Tandem Mass Spectrometry
