# Supplementary material for: Ultrasound-Assisted Recovery and Biological Evaluation of an Astaxanthin-Containing Extract from Chara corallina
Source: Antioxidants (Basel). 2026 Sep 11;15(9):1159. doi: 10.3390/antiox15091159 (PMC13603215; doi:10.3390/antiox15091159)
Supplement: Supplementary file 1 [file antioxidants-15-01159-s001.zip › antioxidants-4514661-supplementary.pdf]

Supplementary Materials

Table S1. Primer sequences used for one-step reverse transcription quantitative PCR (RT-qPCR). Primer specificity was verified using NCBI Primer-BLAST against the corresponding human or mouse RefSeq mRNA sequences. Gene symbols follow the official nomenclature of the HUGO Gene Nomenclature Committee (HGNC) for human genes and Mouse Genome Informatics (MGI) for mouse genes.

**Gene**	**RefSeq**	**Forward primer (5′→3′)**	**Reverse primer (5′→3′)**	**Product (bp)**
Human dermal fibroblasts (HDF)				
SOD1	NM_000454	TGTGACTGCTGACAAAGATG	TCATCTGCTTTTTCATGGAC	117
COL1A2	NM_000089	GAGCACGCTTACTACCTTCA	GCAAGCCATGTATCTTTCAG	105
CAT	NM_001752	TAACTGGGATCTCGTTGGAA	AGGACGTAGGCTCCAGAAGT	148
GPX1	NM_000581	GACTACACCCAGATGAACGA	CGTACTTGAGGGAATTCAGA	133
GAPDH	NM_002046	CGAGATCCCTCCAAAATCAA	TGTGGTCATGAGTCCTTCCA	150
RAW264.7 macrophages				
Nos2	NM_010927	ATCTGGATCAGGAACCTGAA	CCTTTTTTGCCCCCATAGGAA	180
Ptgs2	NM_011198	GGAGAGACTATCAAGATAGTGATC	ATGGTCAGTAGACTTTTACAGCTC	172
Pparg	NM_011146	CAAAGTAGAACCTGCATCTCC	CCTTCACAAGCATGAACTCC	164
Gapdh	NM_008084	AACATCATCCCTGCATCCAC	AGTGGGAGTTGCTGTTGAAG	104

Note: Primer sequences were adopted from previously published studies and subsequently verified for target specificity using NCBI Primer-BLAST against the corresponding RefSeq mRNA sequences. All primer sequences generated a single melting peak and the expected amplicon size under the PCR conditions used in this study.

Table S2. RT-qPCR assay information.

**Gene**	**Slope**	**Efficiency (%)**	**R^2**	**Melt peak (°C)**
SOD1	-3.37	98.1	0.999	82.5
COL1A2	-3.34	99.3	0.998	84.0
CAT	-3.32	100.2	0.999	83.8
GPX1	-3.35	98.8	0.997	81.9
GAPDH	-3.31	100.5	0.999	80.6
Nos2	-3.36	98.4	0.998	84.7
Ptgs2	-3.33	99.7	0.999	85.3
Pparg	-3.35	98.6	0.998	83.5
Gapdh	-3.32	100.0	0.999	81.2

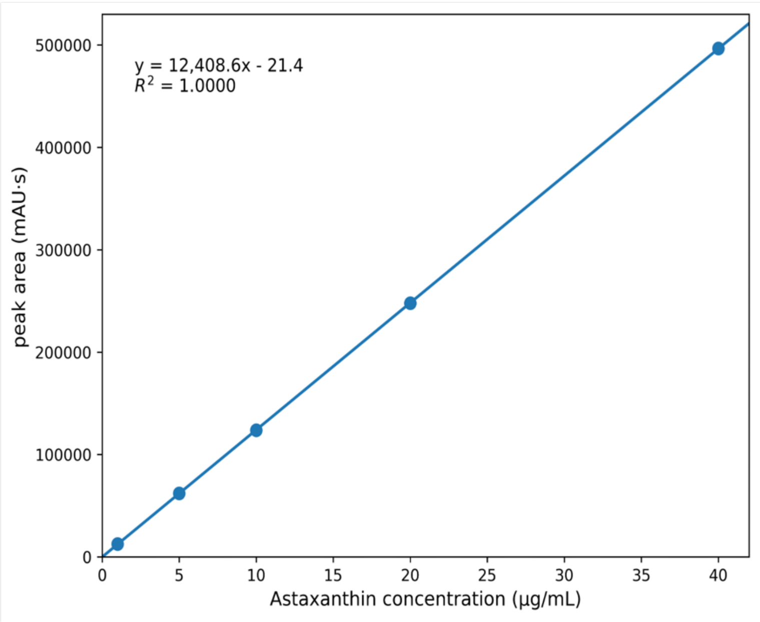

Figure S1. Calibration curve of the authentic astaxanthin standard analyzed by HPLC–PDA at 476 nm. The calibration curve was constructed by plotting chromatographic peak area against astaxanthin concentration over the range of 1–40 µg mL^{-1}. Linear regression yielded the equation y = 12,408.6x − 21.4, with a coefficient of determination of R^2 = 1.0000.
